# Seasonality in spatial distribution: Climate and land use have contrasting effects on the species richness of breeding and wintering birds

**DOI:** 10.1002/ece3.5286

**Published:** 2019-06-20

**Authors:** Kazuhiro Kawamura, Yuichi Yamaura, Masayuki Senzaki, Mutsuyuki Ueta, Futoshi Nakamura

**Affiliations:** ^1^ Department of Forest Science, Graduate School of Agriculture Hokkaido University Hokkaido Japan; ^2^ Department of Forest Vegetation Forestry and Forest Products Research Institute Tsukuba Japan; ^3^ Fenner School of Environment and Society Australian National University Canberra Australian Capital Territory Australia; ^4^ Shikoku Research Center Forestry and Forest Products Research Institute Asakuranishi Kochi Japan; ^5^ Biodiversity Assessment and Projection Section, Center for Environmental Biology and Ecosystem Studies National Institute for Environmental Studies Tsukuba City Japan; ^6^ Faculty of Environmental Earth Science Hokkaido University Sapporo Hokkaido Japan; ^7^ Japan Bird Research Association Tokyo Japan

**Keywords:** forest birds, grassland birds, migration, snow depth, surrounding habitat, temperature

## Abstract

**Aim:**

Many studies have examined large‐scale distributions of various taxa and their drivers, emphasizing the importance of climate, topography, and land use. Most studies have dealt with distributions over a single season or annually without considering seasonality. However, animal distributions and their drivers can differ among seasons because many animals migrate to suitable climates and areas with abundant prey resources. We aim to clarify seasonality in bird distributions and their drivers.

**Location:**

Japan.

**Methods:**

We examined the effects of climate (annual mean temperature, snow depth), topography (elevation), and land use (extent of surrounding habitat) on bird species richness, in the breeding and wintering seasons separately, using nationwide data (254 forest and 43 grassland sites, respectively). We separately analyzed the species richness of all species, residents, short‐, and long‐distance migrants in forests and grasslands.

**Results:**

In the breeding season, the annual mean temperature negatively affected all groups (except for forest and grassland residents), and the extent of surrounding habitat positively affected many groups. By contrast, in the wintering season, temperature positively affected all groups (except for forest residents), and the extent of surrounding habitat positively affected only grassland long‐distance migrants. In both seasons, the species richness of forest and grassland residents was high in regions of moderate and high temperature, respectively. Moreover, snow depth negatively affected all forest groups in the wintering season. Mapping expected species richness suggested that regions with different climates served as habitats for different groups during different seasons.

**Main conclusions:**

All regions were important bird habitats depending on the season, reflecting the contrasting effects of temperature across seasons. In the breeding season, surrounding land use was also an important driver. To understand the seasonal role that each region and environment plays in maintaining species/communities, a large‐scale study considering both environmental seasonality and species distribution is needed.

## 1 INTRODUCTION

Exploration of large‐scale biodiversity patterns and drivers thereof is a fundamental theme of ecology (Gaston, 2000; Wallace, 1876). Many studies have evaluated the relationships between environmental factors and the large‐scale distribution patterns of various taxa (Barbet‐Massin, Thuiller, & Jiguet, 2012; Kreft & Jetz, 2007; Howard, Stephens, Pearce‐Higgins, Gregory, & Willis, 2015). Both climate (e.g., temperature and precipitation) and topography (e.g., elevation) are major drivers of large‐scale organism distributions; these factors determine the productivity of vegetation (Hawkins et al., 2003). Thus, the species richness of various taxa is higher in warm, wet areas with low to moderate altitude (Gaston, 2000; Hawkins et al., 2003). Moreover, recent studies have suggested that land use (e.g., the extent of surrounding appropriate habitat) is also an important driver in this context (Newbold et al., 2016; Yamaura, Amano, Kusumoto, Nagata, & Okabe, 2011).

Most previous studies analyzed the distribution of organisms over a single season (particularly the breeding season) or the annual distribution based on the geographical range maps and did not consider seasonality of either environments or species distributions (Ding, McGill, Fox, & Svenning, 2006; Yamaura et al., 2011; but see Lennon, Greenwood, & Turner, 2000). However, the suitability of various regions as homes for animals can differ seasonally. In cool regions (e.g., areas with deciduous broad‐leaved forests), the productivity of plants and insects increases rapidly from spring to summer (Herrera, 1978; Huston & Wolverton, 2009). For animals using these rapidly increasing resources (termed “prey pulses”), cool regions would be more suitable than other regions at this time (Dalby, McGill, Fox, & Svenning, 2014; Fristoe, 2015; Yamaura et al., 2011). By contrast, regions that are cool in winter may be unsuitable for many animals because of their harsh climate and low productivity (Somveille, Rodrigues, & Manica, 2015).

To cope with such seasonality, many animals engage in seasonal migration (Bauer & Hoye, 2014; Marchand, 2014). For example, most breeding migratory birds, which are important components of bird communities from the cool‐temperate to the arctic zone, move to warmer regions in winter (Kirby et al., 2008; Newton, 2008). Furthermore, the effects of drivers may differ among seasons, and distribution studies across single seasons may overlook regions that exhibit high levels of species richness in other seasons. In winter, snow is present over vast areas from the temperate to the arctic zone, covering about half of the land mass in the Northern Hemisphere (Hori et al., 2017). In grasslands, snow may cover the vegetation completely. Even in forests, some arboreal birds forage on the ground more frequently in the wintering season than in the breeding season, probably because prey is relatively abundant on the ground in winter (Cale, 1994; Hartley, 1987). The availability of vegetation or prey on the ground decreases with increasing snow depth, and this affects the distribution of many animals in winter (Johnson & Sherry, 2001; Leech & Crick, 2007).

The effects of environmental seasonality on species distributions are important because the predicted changes in the global environment may differ among seasons. Increases in temperature due to global warming may be more apparent at higher latitudes, and in winter than in summer (IPCC, 2007, 2014). Furthermore, warmer and lower altitude areas have been altered by human land use; pristine areas only remain in harsh environments (Potapov et al., 2017; Sabatini et al., 2018). Therefore, in this study, we explored the effects of climate (annual mean temperature and maximum snow depth), topography (elevation), and land use (extent of suitable habitat within 1.25, 5, or 10 km of a monitoring site) on bird species richness, in the breeding and wintering seasons separately, using a large monitoring dataset (254 forest and 43 grassland sites evaluated by the same method in both seasons). The sites were distributed across Japan, from the warm‐temperate to the boreal zone (approximately 31–45.5°N). We analyzed the effects of environmental factors on the relative species richness of all species and of three specific groups (residents, short‐distance [SD] migrants, and long‐distance [LD] migrants) in each of two types of habitat (forest and grassland). Finally, to explore the seasonal role played by each region in terms of bird species richness, we separately mapped the expected species richness of each group and of all species as functions of climate, land use, and topography in the breeding and wintering seasons.

## 2 MATERIALS AND METHODS

### 2.1 Bird data

Bird data were collected in a survey of terrestrial birds in Japan; this was a component of the “Monitoring Sites 1,000 Project” conducted by the Ministry of the Environment (http://www.biodic.go.jp/moni1000/findings/data/index_file_terrestrialbird.html [in Japanese]). This nationwide survey commenced in 2003; both forest and grassland sites were visited. The forest sites are principally natural forests (deciduous or evergreen broad‐leaved, evergreen conifer, or mixed forests) and the grasslands include wet and dry grasslands and meadows. At each site, a survey is conducted every 1–5 years. In each survey year, trained volunteers visit each site four times in the breeding (April to July) and wintering (December to February) seasons and record all birds detected within a 50‐m radius of five point‐count locations, which are >100 m apart. The surveys are conducted on both clear and cloudy days (from 0400 to 0900 hr in the breeding season and from 0800 to 1100 hr in the wintering season) that are not very windy. The survey period in the breeding season is earlier in southern areas where migrants first arrive (i.e., from April to May in Kyusyu, from June to July in Hokkaido), and the survey clock time is chosen, in certain regions, to avoiding the buzzing of cicadas (i.e., from 0400 hr in Hokkaido and northern Honshu where cicada buzzing can be very loud, and from 0500 or 0600 hr in other regions).

We used the survey results from 2009 to 2015 because the same survey method (i.e., point census counts) was used in each of these years. To simplify the analyses, we used only the total species richness observed in five point‐count locations for each site in the latest year as the analysis unit. To control for island size effects, we evaluated data from sites on only the four largest islands of the Japanese archipelago (i.e., Honshu, Hokkaido, Kyusyu, and Shikoku; Yamaura et al., 2011; Katayama et al., 2014; Appendix S1). In addition, we excluded one high‐altitude site in Hokkaido (Mt. Daisetsu) because the annual mean temperature there is very low; hence, we regarded the site as an outlier. Ultimately, we used data from 254 forest and 43 grassland sites visited in both breeding and wintering seasons (Appendix S2). We excluded data on nocturnal birds because of mismatches in terms of diurnal survey clock times, and we also excluded data on raptors, which generally have large home ranges and may thus move among adjacent sites (Jetz, Carbone, Fulford, & Brown, 2004).

Based on previous reports (Kiyosu, 1965; Takagawa et al., 2011; Ueta, Fukui, Yamaura, & Yamamoto, 2011; Yamaura et al., 2011), we categorized all bird species into three groups by migratory traits (LD migrants, SD migrants, and residents) and two groups by habitat preference (forest and grassland, i.e., generalists were also categorized into either group; Appendix S3). Next, we determined the relative species richness of all species and of each group (residents, SD migrants, and LD migrants) during each season at each site. We evaluated only species inhabiting principally forests at the forest sites and only species inhabiting principally grasslands at the grassland sites. We defined LD migrants as species migrating from outside Japan to breed or overwinter in Japan, SD migrants as species migrating within Japan to breed and overwinter in different regions, and residents as species that are relatively sedentary throughout the year (Yamaura et al., 2009).

### 2.2 Environmental factors

Environmental factors that potentially affect bird species richness include the annual mean temperature and the maximum snow depth (climatic factors; Leech & Crick, 2007; Yamaura et al., 2011), elevation (a topographic factor; Davies et al., 2007), and the extent of appropriate surrounding habitat within 1.25, 5, or 10 km of the center of each site (a land‐use factor; Barbet‐Massin, Thuiller, & Jiguet, 2012; Howard et al., 2015). We calculated the value of each environmental factor at the center of each site (the centroid of the five point‐count locations at each site) using ArcGIS 10.2.2 software (ESRI).

#### 2.2.1 Climate

The annual mean temperature is important in terms of breeding bird species richness in Japan (Yamaura et al., 2011). In high‐rainfall regions such as Japan, temperature determines vegetation productivity and seasonality (Hawkins et al., 2003; Ichii, Kawabata, & Yamaguchi, 2002; Appendix S4), both of which are important for bird distributions (Fristoe, 2015; Hurlbert & Haskell, 2003; Somveille et al., 2015). We found that the annual mean temperature was strongly correlated with the mean temperature during the breeding/wintering season, suggesting that considering these variables simultaneously in analyses would be problematic. Therefore, to compare the effects of these factors, we constructed generalized linear mixed models (GLMMs) for preliminary analyses. The results showed that, for both forests and grasslands, the annual mean temperature performed well in both seasons (i.e., for six groups this showed the lowest Akaike information criterion [AIC], for another seven groups the ΔAIC was <2, and for the remaining three groups the ΔAIC was < 3; Appendix S5). Therefore, we selected the annual mean temperature for all analyses.

In the wintering season, snow cover can negatively affect bird survival and prey availability near the ground (Leech & Crick, 2007). Therefore, we selected the maximum snow depth (hereinafter: snow depth) as an additional climatic factor when analyzing data from the wintering season. We used the Mesh Climate Value 2010 (a contiguous nationwide grid of 1‐km^2^ squares with 30‐year [1981–2010] mean monthly temperatures, snow depths, and other data provided by the Meteorological Agency of Japan; Appendix S6a,b; http://nlftp.mlit.go.jp/ksj/index.html [in Japanese]) to this end.

#### 2.2.2 Topography

Elevation is important in terms of large‐scale bird distributions (Davies et al., 2007). Therefore, we selected elevation as a topographic variable. We used the Elevation and Slope Angle Tertiary Mesh, (a contiguous nationwide grid of 1‐km^2^ squares with mean elevations and other data, provided by the Geospatial Information Authority of Japan; Appendix S6c; http://nlftp.mlit.go.jp/ksj/index.html [in Japanese]) to this end.

#### 2.2.3 Land use

The extent of habitat on the landscape scale is one of the most commonly used explanatory variables in studies on the effects of land use on the large‐scale distributions of organisms (including birds; e.g., Barbet‐Massin et al., 2012; Howard et al., 2015). We used the High Resolution Land‐Use and Land‐Cover Map (2006–2011) of the Japan Aerospace Exploration Agency (JAXA) (Appendix S7; http://www.eorc.jaxa.jp/ALOS/lulc/jlulc_jpn.htm [in Japanese] to this end); these are the latest land‐use data. However, the accuracy of land‐use categorization was low (e.g., the accuracy rate for evergreen broad‐leaved forests was <36% because of incorrect categorization of such forests as other forest types). Although the landscape effects of deciduous broad‐leaved natural forests may differ from those of conifer plantations (Yamaura et al., 2011), we were forced to use the total extent of forests (i.e., evergreen broad‐leaved, deciduous broad‐leaved, evergreen conifer, and deciduous conifer forests) when evaluating forest sites, and the total extent of open habitats (paddy fields, grasslands, and agricultural lands) when evaluating grassland sites. To compare the effects of the extent of habitat (forests for analyses of forest groups and open habitats for analyses of grassland groups) within 1.25, 2.5, 5, 10, and 15 km of the center of each site (hereafter, surrounding habitat), we constructed GLMMs for preliminary analyses. The results showed that, for the analyses of forest groups in the breeding season, the extent of habitat within 1.25 km gave the best quality model (i.e., for all species, residents, and SD migrants this showed the lowest AIC, and for LD migrants the ΔAIC was 0.2). However, the surrounding habitat within 10 km offered the best model for analyses of forest groups in the wintering season (i.e., for SD migrants this showed the lowest AIC, and for all other groups the ΔAIC was <2), and surrounding habitat within 5 km performed best for analyses of grassland groups in both seasons (i.e., for all species in the breeding season and LD migrants in both seasons, this showed the lowest AIC, and for the other groups, except for SD migrants in the breeding season, the ΔAIC was <2; Appendix S8). Therefore, we selected these land‐use factors to analyze each habitat and season.

### 2.3 Statistical analyses

We analyzed the effects of environmental factors on the relative species richness of each group and of all species using a GLMM that considered spatial autocorrelation (a spatial GLMM, Family = Poisson, link function = log; Dormann et al., 2007). This allowed us to evaluate spatial correlation structures using a variance–covariance matrix that reflected the distances between locations, and has been used in previous studies (Brambilla & Ficetola, 2012; Lennon, Beale, Reid, Kent, & Pakeman, 2011). We chose the exponential distribution as the correlation function, based on the semivariograms (Appendix S9; Dormann et al., 2007). For each season (breeding and wintering), we separately considered the relative species richness of all species and that of three bird groups (residents, SD migrants, and LD migrants) in two habitats (forest and grassland) as response variables.

To avoid multicollinearity, we calculated variance inflation factors (VIFs) before the analyses, using models featuring linear values for each environmental factor for all species in each habitat and season. For grassland in the wintering season, we found that the VIF of the annual mean temperature was >3; however, we considered temperature a particularly important driver in both seasons. Therefore, we removed snow depth, which had the second largest VIF, from our analyses of grassland birds in the wintering season. Finally, in both habitats in both seasons, the VIF for every variable was <3, suggesting that all variables were suitable (Zurr, Ieno, & Elphick, 2010; Appendix S10). We evaluated six explanatory variables (annual mean temperature, the extent of surrounding habitat, and elevation and their squares) that might affect both breeding forest birds and breeding and wintering grassland birds, and eight explanatory variables (these three factors and snow depth, and their squares) that might influence wintering forest birds. All environmental factors were standardized prior to analyses.

The spatial GLMM did not allow calculation of the AIC because the algorithm exploits the penalized quasi‐likelihood method. Thus, to select the most parsimonious model (the best spatial model), we performed backward selection based on the semi‐partial *R* ^2^ of the explanatory variables (Jaeger, Edwards, Das, & Sen, 2017). First, we created a full model featuring all explanatory variables. Then, we deleted only one insignificant variable (least semi‐partial *R* ^2^), and repeated this step until only significant variables (semi‐partial *R* ^2^ > 0.02, and lower limit 95% confidence interval for semi‐partial *R* ^2^ > 0.001) remained. We conducted all analyses using the *glmmPQL* function of “MASS” v. 7.3.45 (Venables & Ripley, 2002), “nlme” v. 3.1.131 (Pinheiro, Bates, DebRoy, & Sarkar, 2017), and the *r2beta* function of “r2glmm” v. 0.1.2. (Jaeger, 2017) running in R v. 3.3.0 (R core team, 2016).

To determine whether we could use the spatial GLMMs (assuming a Poisson distribution without considering over‐dispersion and survey year effects), we constructed generalized linear models (GLMs assuming a Poisson distribution), GLMMs with random site effects to account for over‐dispersion, and GLMMs with random year effects. Coefficients obtained from the GLMs and the three types of GLMMs were nearly identical, and the coefficients of the factors involved in the best spatial models never varied by more than 15%, suggesting that considering the effects of over‐dispersion and survey years is unnecessary. Furthermore, when we changed the value of the semi‐partial *R* ^2^ for model selection from 0.02 to 0.05 or 0.1, the main results did not change.

### 2.4 Mapping bird species richness

We mapped the expected species richness of all species and of three bird groups (all species, residents, SD migrants, and LD migrants) found in two habitats (forest and grassland). We divided the four largest islands of Japan into grid squares of 1 km in length. This was the same grid size that we used for the climate and topography data. Then, we calculated the values of environmental factors (annual mean temperature, snow depth, elevation, and extent of surrounding habitat within 1.25, 5, and 10 km) at the center of each grid. Finally, we mapped the expected species richness and any differences among seasons on the grid scale, using the best (i.e., most parsimonious) spatial models derived from backward selection (Table 1). In this context, we assumed that the areas used for predictions were covered by homogenous forests or grasslands (i.e., forest sites were assumed to be natural forests, as were the survey sites). We used *ArcGIS 10.2.2* (ESRI, CA) to perform all mapping procedures.

**Table 1:** The best spatial GLMMs (estimates ± standard errors [semi‐partial *R* ^2^, Wald *p*‐value])

Variables	All grassland species		All forest species	
	Breeding (*R* ^2^ = 0.377)	Wintering (*R* ^2^ = 0.688)	Breeding (*R* ^2^ = 0.173)	Wintering (*R* ^2^ = 0.198)
Intercept	2.15 ± 0.08	1.52 ± 0.16	2.82 ± 0.02	2.56 ± 0.03
TEMP	−0.13 ± 0.04 (*R* ^2^ = 0.203, *p* < 0.01)	0.99 ± 0.16 (*R* ^2^ = 0.672, *p* < 0.01)	−0.08 ± 0.02 (*R* ^2^ = 0.068, *p* < 0.01)	
(TEMP)^2^	0.15 ± 0.06 (*R* ^2^ = 0.142, *p* = 0.02)	−0.35 ± 0.16 (*R* ^2^ = 0.112, *p* = 0.03)	−0.05 ± 0.02 (*R* ^2^ = 0.050, *p* < 0.01)	−0.06 ± 0.02 (*R* ^2^ = 0.039, *p* < 0.01)
SNOW	×	×	×	
(SNOW)^2^	×	×	×	−0.06 ± 0.01 (*R* ^2^ = 0.126, *p* < 0.01)
ELEV				
(ELEV)^2^				
AREA	0.13 ± 0.05 (*R* ^2^ = 0.175, *p* = 0.01)		0.06 ± 0.02 (*R* ^2^ = 0.049, *p* < 0.01)	
(AREA)^2^	−0.08 ± 0.04 (*R* ^2^ = 0.089, *p* = 0.07)			
Range	3.666 km	16.091 km	0.001 km	1.046 km

Variables	Grassland residents		Forest residents	
	Breeding (*R* ^2^ = 0.638)	Wintering (*R* ^2^ = 0.424)	Breeding (*R* ^2^ = 0.136)	Wintering (*R* ^2^ = 0.082)
Intercept	0.37 ± 0.15	−0.67 ± 0.24	1.89 ± 0.02	1.90 ± 0.02
TEMP	0.78 ± 0.14 (*R* ^2^ = 0.614, *p* < 0.01)	1.10 ± 0.22 (*R* ^2^ = 0.424, *p* < 0.01)		−0.07 ± 0.02 (*R* ^2^ = 0.072, *p* < 0.01)
(TEMP)^2^	−0.31 ± 0.13 (*R* ^2^ = 0.122, *p* = 0.02)		−0.06 ± 0.02 (*R* ^2^ = 0.068, *p* < 0.01)	
SNOW	×	×	×	
(SNOW)^2^	×	×	×	−0.04 ± 0.01 (*R* ^2^ = 0.039, *p* < 0.01)
ELEV				
(ELEV)^2^	0.12 ± 0.06 (*R* ^2^ = 0.114, *p* = 0.08)		0.03 ± 0.01 (*R* ^2^ = 0.041, *p* < 0.01)	
AREA			0.06 ± 0.02 (*R* ^2^ = 0.026, *p* < 0.01)	
(AREA)^2^				
Range	0.000 km	0.001 km	0.001 km	2.546 km

Variables	Grassland SD migrants		Forest SD migrants	
	Breeding (*R* ^2^ = 0.191)	Wintering (*R* ^2^ = 0.627)	Breeding (*R* ^2^ = 0.129)	Wintering (*R* ^2^ = 0.405)
Intercept	1.44 ± 0.09	0.93 ± 0.19	1.72 ± 0.03	1.59 ± 0.04
TEMP	−0.12 ± 0.05 (*R* ^2^ = 0.135, *p* = 0.02)	0.77 ± 0.25 (*R* ^2^ = 0.605, *p* < 0.01)	−0.08 ± 0.02 (*R* ^2^ = 0.053, *p* < 0.01)	0.25 ± 0.04 (*R* ^2^ = 0.178, *p* < 0.01)
(TEMP)^2^	0.12 ± 0.07 (*R* ^2^ = 0.069, *p* = 0.10)	−0.29 ± 0.17 (*R* ^2^ = 0.109, *p* = 0.03)	−0.04 ± 0.02 (*R* ^2^ = 0.021, *p* = 0.02)	
SNOW	×	×	×	
(SNOW)^2^	×	×	×	−0.08 ± 0.02 (*R* ^2^ = 0.134, *p* < 0.01)
ELEV				0.12 ± 0.04 (*R* ^2^ = 0.038, *p* < 0.01)
(ELEV)^2^				−0.07 ± 0.03 (*R* ^2^ = 0.030, *p* = 0.02)
AREA				
(AREA)^2^			−0.04 ± 0.01 (*R* ^2^ = 0.051, *p* < 0.01)	
Range	0.017 km	9.904 km	0.000 km	1.320 km

Variables	Grassland LD migrants		Forest LD migrants	
	Breeding (*R* ^2^ = 0.554)	Wintering (*R* ^2^ = 0.493)	Breeding (*R* ^2^ = 0.167)	Wintering (*R* ^2^ = 0.194)
Intercept	0.93 ± 0.17	−0.10 ± 0.25	1.56 ± 0.04	0.62 ± 0.06
TEMP	−0.46 ± 0.08 (*R* ^2^ = 0.446, *p* < 0.01)	0.74 ± 0.20 (*R* ^2^ = 0.359, *p* < 0.01)	−0.18 ± 0.03 (*R* ^2^ = 0.110, *p* < 0.01)	0.13 ± 0.05 (*R* ^2^ = 0.023, *p* = 0.02)
(TEMP)^2^	0.22 ± 0.13 (*R* ^2^ = 0.082, *p* = 0.10)		−0.08 ± 0.03 (*R* ^2^ = 0.039, *p* < 0.01)	−0.10 ± 0.05 (*R* ^2^ = 0.023, *p* = 0.04)
SNOW	×	×	×	−0.10 ± 0.04 (*R* ^2^ = 0.073, *p* = 0.01)
(SNOW)^2^	×	×	×	
ELEV		0.55 ± 0.28 (*R* ^2^ = 0.078, *p* = 0.06)		
(ELEV)^2^		−0.29 ± 0.23 (*R* ^2^ = 0.084, *p* = 0.21)		
AREA	0.38 ± 0.10 (*R* ^2^ = 0.316, *p* < 0.01)	0.37 ± 0.13 (*R* ^2^ = 0.113, *p* < 0.01)	0.07 ± 0.03 (*R* ^2^ = 0.021, *p* = 0.03)	
(AREA)^2^	−0.19 ± 0.08 (*R* ^2^ = 0.124, *p* = 0.03)			
Range	0.148 km	12.093 km	0.000 km	0.000 km

Abbreviations: AREA, area of surrounding suitable habitat (within a radius of 1.25 km for breeding forest birds, 10 km for wintering forest birds, and 5 km for grassland birds in both seasons); Breeding, breeding season; Cross marks, explanatory variables that were not used in construction of the full model to avoid multicollinearity; ELEV, elevation; LD, long distance; Range, the average distance over which values for the response variable are correlated (note that spatial autocorrelation must be considered when these distances are greater than the minimum distance between survey sites [forest sites: 0.537 km, grassland sites: 14.648 km]); SD, short distance; SNOW, maximum snow depth; TEMP, annual mean temperature; Wintering: wintering season.

## 3 RESULTS

In the breeding season, the best model for all three groups (residents, SD migrants, and LD migrants) and all species in both habitats included annual mean temperature, which had highest explanatory power. For four of the six groups (LD migrants in both habitats, and forest residents and SD migrants) and all species in both habitats, the extent of surrounding habitat was also included in the model (Table 1). In the wintering season, the best model for all three forest groups and all forest species included annual mean temperature and snow depth (Table 1), and the best models for all three grassland groups and all grassland species also included temperature. Variations in species richness were substantially explained by these climatic factors alone. For all groups (except residents in both habitats), the explanatory power of the wintering season models was comparable to, or higher than, those of breeding birds (Table 1).

### 3.1 Effects of annual mean temperature

**Figure 1:**
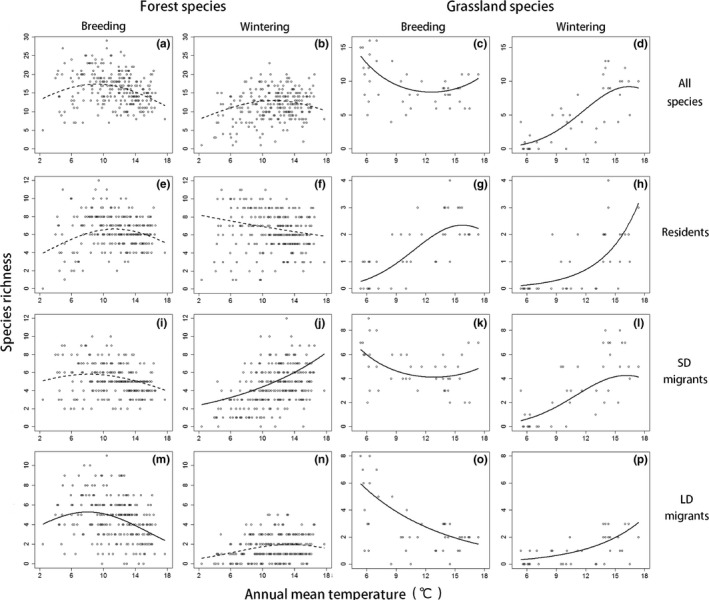
Relationships between annual mean temperature and the species richness of forest birds (a, e, i, m: breeding season; b, f, j, n: wintering season) and grassland birds (c, g, k, o: breeding season, d, h, l, p: wintering season). Data reflecting migratory traits are shown in each line of the Figure (a–d: all species; e–h: residents; i–l: short‐distance migrants; m–p: long‐distance migrants). Dots indicate data collected at each site. Lines represent the estimates afforded by the best spatial models (solid lines indicate semi‐partial *R* ^2^ of either the temperature and its squared term >0.1, and broken lines indicate that of both the temperature and its squared term <0.1). We used a GLMM (a multivariate analysis), and the effects of the other explanatory variables were considered and fixed to mean values in each figure. See details of the best spatial models in Table 1. Abbreviations: Breeding, breeding season; LD, long distance; SD, short distance; Wintering, wintering season

During the breeding season, for SD migrants and LD migrants from both forests and grasslands, species richness was high in areas of low annual mean temperature (Figure 1i,k,m,o), although the pattern shown by forest SD migrants was less pronounced than patterns shown by other groups (Figure 1i). Reflecting these patterns, in cool areas, the species richness of all grassland species was high and that of all forest species also tended to be high (Figure 1a,c). The species richness of forest residents was relatively higher in areas of moderate temperature (approximately 10°C), although this pattern was not obvious (Figure 1e). By contrast, the species richness of grassland residents was higher in warmer areas (Figure 1g).

By contrast, in the wintering season, for both forest and grassland birds in SD migrant and LD migrant groups, species richness was greater in areas of higher annual mean temperature (Figure 1j,l,n,p), although the patterns of forest LD migrants were less clear than those of other groups (Figure 1n). Reflecting these patterns, in warm areas, the species richness of all grassland species was high and that of all forest species also tended to be high (Figure 1b,d). The positive effects of temperature on the diversity of grassland species were more notable than the effect on forest species. For forest residents, species richness tended to be low in warm areas (Figure 1f). Grassland residents exhibited patterns similar to those in the breeding season; the species richness was greater in warmer areas (Figure 1h).

### 3.2 Effects of snow depth

**Figure 2:**
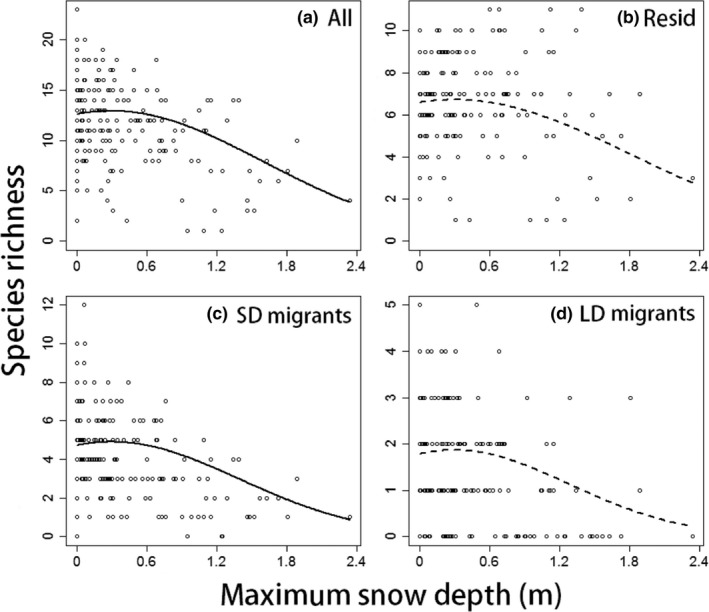
Relationships between maximum snow depth and the species richness of wintering forest bird species (a) all species, (b) residents, (c) short‐distance migrants, (d) long‐distance migrants. Dots indicate data derived at each site; lines reflect the estimates of the best spatial models (solid lines indicate semi‐partial *R* ^2^ of either the temperature and its squared term >0.1, and broken lines indicate that of both the temperature and its squared term <0.1). We used a GLMM (a multivariate analysis), and the effects of the other explanatory variables were considered and fixed to mean values in each figure. See details of the best spatial models in Table 1. Abbreviations: LD, long distance; Resid, residents; SD, short distance

In the wintering season, in areas with deeper snow, the species richness of forest SD migrants was lower, and that of forest residents and LD migrants also tended to be lower (Figure 2b–d). In such areas, the species richness of all species was lower, reflecting the patterns of individual groups (Figure 2a).

### 3.3 Effects of the extent of surrounding habitat

**Figure 3:**
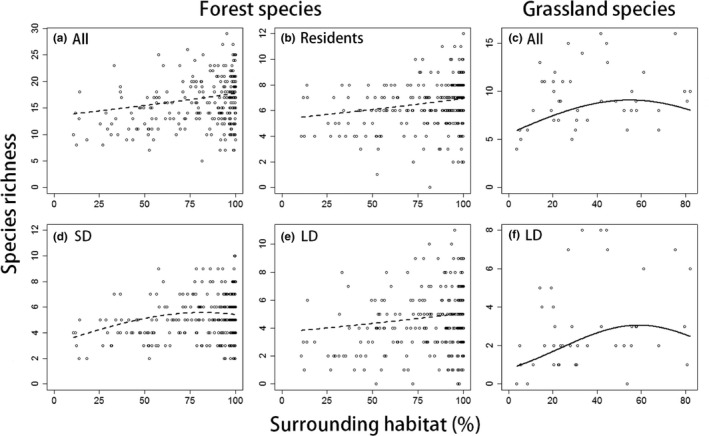
Relationships between the extent of surrounding habitat and the species richness of breeding forest birds (a) all species, (b) residents, (d) short‐distance migrants, (e) long‐distance migrants and breeding grassland birds (c) all species, (f) long‐distance migrants. Dots indicate data derived at each site. Lines reflect the estimates afforded by the best spatial models (solid lines indicate semi‐partial *R* ^2^ of either the temperature and its squared term >0.1, and broken lines indicate that of both the temperature and its squared term <0.1). We used a GLMM (a multivariate analysis), and the effects of the other explanatory variables were considered and fixed to mean values in each figure. See details of the best spatial models in Table 1. Abbreviations: All, all species; LD, long‐distance migrants; SD, short‐distance migrants; Surrounding habitat: the extent of surrounding habitat within 1.25 km (a, b, d, e) and 5 km (c, f)

In the breeding season, for all three forest groups (residents, SD migrants, and LD migrants) and one grassland group (LD migrants), species richness tended to be high in areas with large extents of surrounding habitat, although these effects were small for all groups except grassland LD migrants (Figure 3b,d–f). Reflecting these patterns, in areas with large extents of surrounding habitat, the species richness of all grassland species was high and that of all forest species also tended to be high (Figure 3a,c). By contrast, during the wintering season, the species richness of only one group (grassland LD migrants) was higher in areas with larger extents of surrounding habitat (Appendix S11). No effects of the extent of surrounding habitat were evident in the other five groups or in all species in both habitats (Table 1).

### 3.4 Effects of elevation

There were no obvious effects of elevation in either season. In the breeding season, the species richness of residents in both habitats was relatively high in areas of markedly high elevation (Appendix S12a,b). In the wintering season, unimodal relationships between species richness and elevation were observed for forest SD and grassland LD migrants (Appendix S12c,d).

### 3.5 Mapping predicted bird species richness

**Figure 4:**
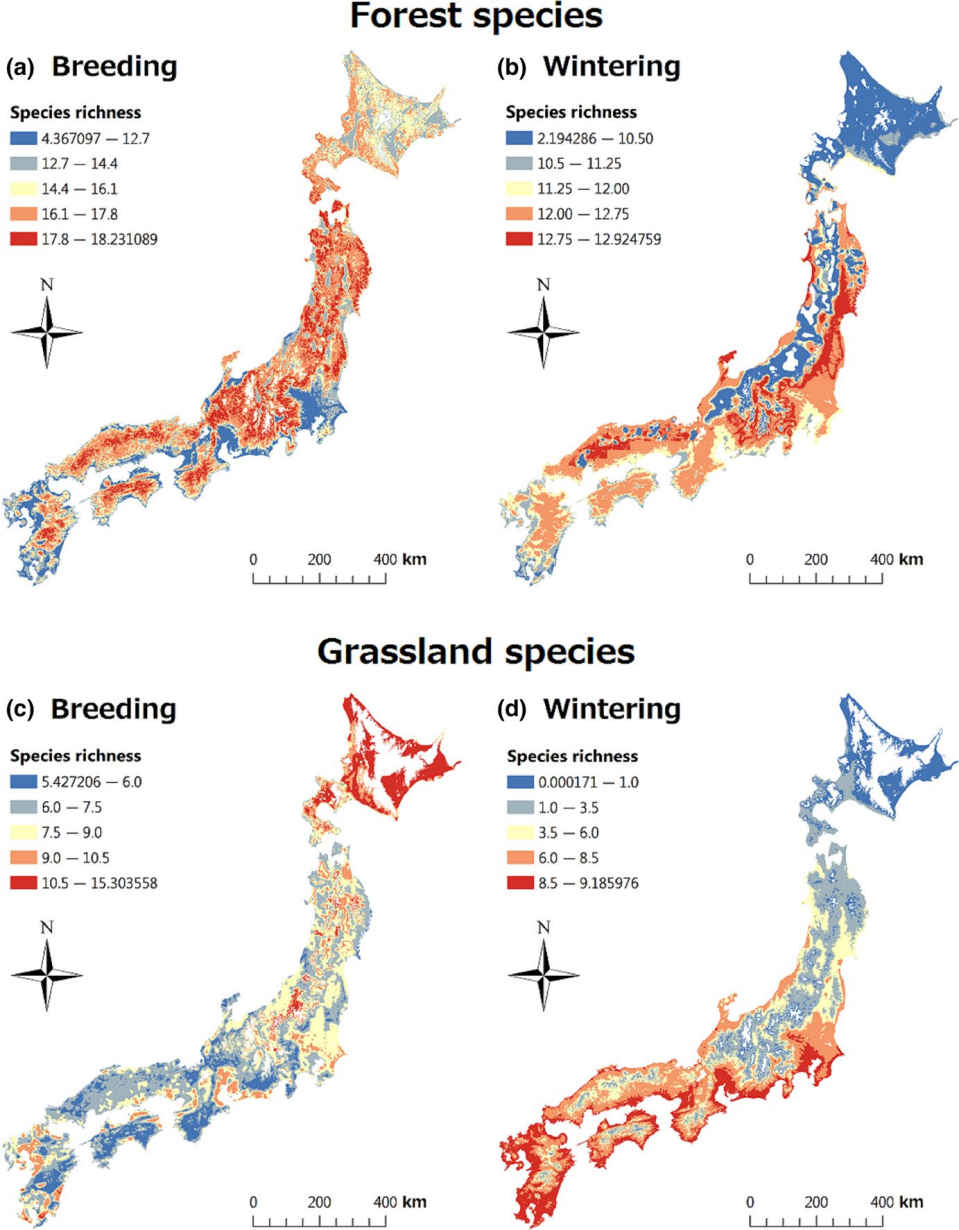
The expected species richness of (a,b) forest birds and (c,d) grassland birds. Breeding season (a, c); wintering season (b, d). We used the best spatial models to derive these results. We excluded regions where the values of explanatory variables from the models were not within the analysis range (i.e., annual mean temperature <2°C in forests, annual mean temperature <5°C in grasslands, elevation >1,600 m, snow depth > 2.4 m). Coordinates: 30°59′–45°31′N; 129°33′–145°49′E

Reflecting the contrasting effects of climate on the six groups (all species, SD migrants, and LD migrants of both forests and grasslands), we found that regions exhibiting high species richness differed between the breeding and wintering seasons. Specifically, in the breeding season, the species richness of the six groups was high in northern Japan, and the species richness of the three forest groups was also high in the mountains of southern Japan (Figure 4a,c; Appendices S13c,e, S14c,e, S15a,c,d, and S16a,c,d). By contrast, in the wintering season, species richness was high in southern Japan, particularly in the plains near the Pacific coast (Figure 4b,d; Appendices S13d,f, S14d,f, S15a,c,d, and S16a,c,d).

Unlike the situation with migrants, the regions exhibiting high resident species richness of both habitats were similar in the two seasons. Therefore, the species richness of grassland residents was high in southern Japan, particularly in the plains near the Pacific coast. This was also the case for wintering migrants (Appendices S14a,b and S16b). The species richness of forest residents was high in both central Japan and inland regions of southern Japan, which have moderate annual mean temperatures; species richness was also high on the Pacific side of northern Japan during the wintering season (Appendices S13a,b and S15b).

## 4 DISCUSSION

Previous studies on the migration of single species and the distribution of species richness on continental/global scales have shown the importance of seasonality in both bird distribution and environments (Newton, 2008; Somveille et al., 2015). However, there have been few of these studies, and the seasonal effects of environmental factors on the distribution of bird communities at a national level are poorly understood. Using data from systematic field surveys across Japan, we analyzed the seasonal distribution of terrestrial birds, including migrant species. The drivers important for bird distribution differed among seasons. In the breeding season, the species richness of many groups was high in cool areas with large expanses of surrounding habitat. By contrast, during the wintering season, the species richness of all groups (except for forest residents) was high in warm areas. The extent of surrounding habitat positively affected only grassland LD migrants. Moreover, snow depth negatively affected all forest groups. These results suggest that it is important to consider seasonality in both bird distributions and environments even in Japan, a country located in the temperate to boreal zone.

### 4.1 Effects of annual mean temperature

For all species, SD migrants, and LD migrants in both forests and grasslands, the species richness during the breeding season tended to be high in cool regions (Figure 1a,c,i,k,m,o). This is explained by the abundance of prey during the breeding season in cool areas, particularly in deciduous broad‐leaved forests, although few empirical studies have been performed (Appendix S7a; Herrera, 1978; Huston & Wolverton, 2009). In addition, competition may affect the patterns observed. Thus, in cool areas with few bird residents (Figure 1e,g,h) and with abundant prey resources during the breeding season, breeding migrants can use prey not consumed by residents (Fristoe, 2015; Somveille, Rodrigues, & Manica, 2015, 2018). It is also possible that high temperature reduced species richness by having negative physiological effects, such as reductions in egg viability (Cooper, Hochachka, Phillips, & Dhondt, 2006). Importantly, any negative effects of temperature would be greater in grasslands, which lack any shading from sunshine (M. Yui, personal communication).

In contrast to the case in the breeding season, the annual mean temperature tended to positively affect the species richness of all species, SD migrants, and LD migrants in both forests and grasslands in the wintering season (Figure 1b,d,j,l,n,p). This is because most wintering birds avoid the harsh climate and scarce prey resources characteristic of areas of low temperature (Somveille et al., 2015; Somveille, Rodrigues, & Manica, 2018). The positive effects of temperature may be more pronounced in grasslands, which lack shelter from the wind, rain, and snow. In addition, distribution patterns shown by forest groups may also be explained indirectly as effects of vegetation; warm areas dominated by evergreen broad‐leaved forests and conifer plantations may provide more prey resources during the wintering season than cool areas dominated by deciduous broad‐leaved forests (Appendix S7a; Kira, 1991; Yamaura et al., 2011; Huston & Wolverton, 2009), in contrast to the situation in the breeding season.

Unlike the situation for migrants, the species richness of forest residents was relatively high in both seasons in areas of moderate annual mean temperature (Figure 1e,f). Such areas may yield constant amounts of prey and afford a reasonably comfortable climate throughout the year, and thus are conveniently occupied by highly sedentary residents. Although in the wintering season the species richness of forest residents was also high in cooler areas, sampling a larger number of cool areas may show the same patterns that were observed in the breeding season (i.e., unimodal relationships). In both seasons, the species richness of grassland residents was higher in warmer areas (Figure 1g,h), as was also true of wintering migrants, suggesting that the costs imposed by wintering are greater than the benefits obtained during the breeding season in cool areas.

### 4.2 Effects of snow depth

Snow depth tended to negatively affect the species richness of all groups of wintering forest birds (Figure 2), suggesting that snow cover restricted the distributions of various species, including arboreal species. When analyzing grassland birds, we did not use snow depth as an explanatory variable because a strong negative correlation was evident between snow depth and annual mean temperature. However, not only temperature but also snow cover could explain the poor species richness of grassland species in cooler areas (Figure 1d,h,l,p). Although, in some cases, the effects of snow cover can be estimated using temperature data, snow cover would be a major driver of wintering bird distribution in studies that include areas where snowfalls were so heavy that foraging near the ground was restricted.

### 4.3 Effects of the extent of surrounding habitat

We found small but positive effects of the extent of surrounding habitat on the species richness of many groups, usually only in the breeding season (for three groups only in the breeding season [forest residents, SD migrants, and LD migrants] and one group in both seasons [grassland LD migrants]; Figure 3 and Table 1). For all species in both habitats, species richness tended to be high in areas with a large extent of surrounding habitat only in the breeding season. As surrounding habitat affords mating opportunities and the food resources required for breeding (Dale, 2001; Dunning, Danielson, & Pulliam, 1992), the positive effects of a greater extent of surrounding habitat would be larger in the breeding than the wintering season (Yahner, 2000). In some groups, the explanatory power of the breeding season model was considerably less than that of the wintering season model. Therefore, in the breeding season in particular, small‐scale factors (e.g., patch area and configuration, and habitat structure) may account for unexplained variation in species richness, although the extent of surrounding habitat does correlate with some of these variables (Fahrig, 2003). Alternatively, by dividing forests into natural forests and plantation forests, more apparent landscape effects may be detected (Yamaura et al., 2011).

### 4.4 Seasonality in the spatial distributions of species richness

We identified only a few areas exhibiting high‐level species richness throughout the year (Figure 4). Rather, regions with different climates, as dictated by annual mean temperature and snow depth, likely served as habitats for different groups in different seasons; every region surveyed played an important role as a breeding or a wintering bird habitat, contributing to the maintenance of bird diversity in Japan. Bird communities in regions that were cool in the breeding season were probably supported by migrants that overwintered in warm regions with little snow. These warm regions have few old‐growth forests and wetlands that are restricted to cool or high‐altitude areas, untouched by humans (Kusumoto et al., 2017; Yamaura et al., 2011). However, climate and land use in warm areas are important and can affect breeding bird communities via migration. Conversely, these environments in cool areas would also affect wintering bird communities via migration. The explained variation in species richness was limited, especially in forests, suggesting that habitat quality was important. Future studies should examine the effects of local factors at a large scale. Identifying large‐scale seasonal differences in animal distributions and investigating the associated drivers is essential to understanding the maintenance of biodiversity and ensuring its conservation.

## CONFLICT OF INTEREST

None declared.

## AUTHOR CONTRIBUTIONS

KK, YY, and FN conceived the ideas; KK and MU performed the analyses; KK, YY, and MS led the writing. All authors read and commented on the manuscript.

## Data Availability

The data of survey site distribution, bird species richness, and environment are available at Dryad Digital Repository: https://doi.org/10.5061/dryad.7n404q0.
