# Supplementary material for: Seasonality in spatial distribution: Climate and land use have contrasting effects on the species richness of breeding and wintering birds
Source: Ecol Evol. 2019 Jun 20;9(13):7549–61. doi: 10.1002/ece3.5286 (PMC6636198; doi:10.1002/ece3.5286)
Supplement: Supplementary file 1 [file ECE3-9-7549-s001.docx]

*Ecology & Evolution*

**SUPPORTING INFORMATION**

**Seasonality in spatial distribution: Climate and land-use have contrasting effects on the species richness of breeding and wintering birds**

Kazuhiro KAWAMURA, Yuichi YAMAURA, Masayuki SENZAKI, Mutsuyuki UETA, Futoshi NAKAMURA


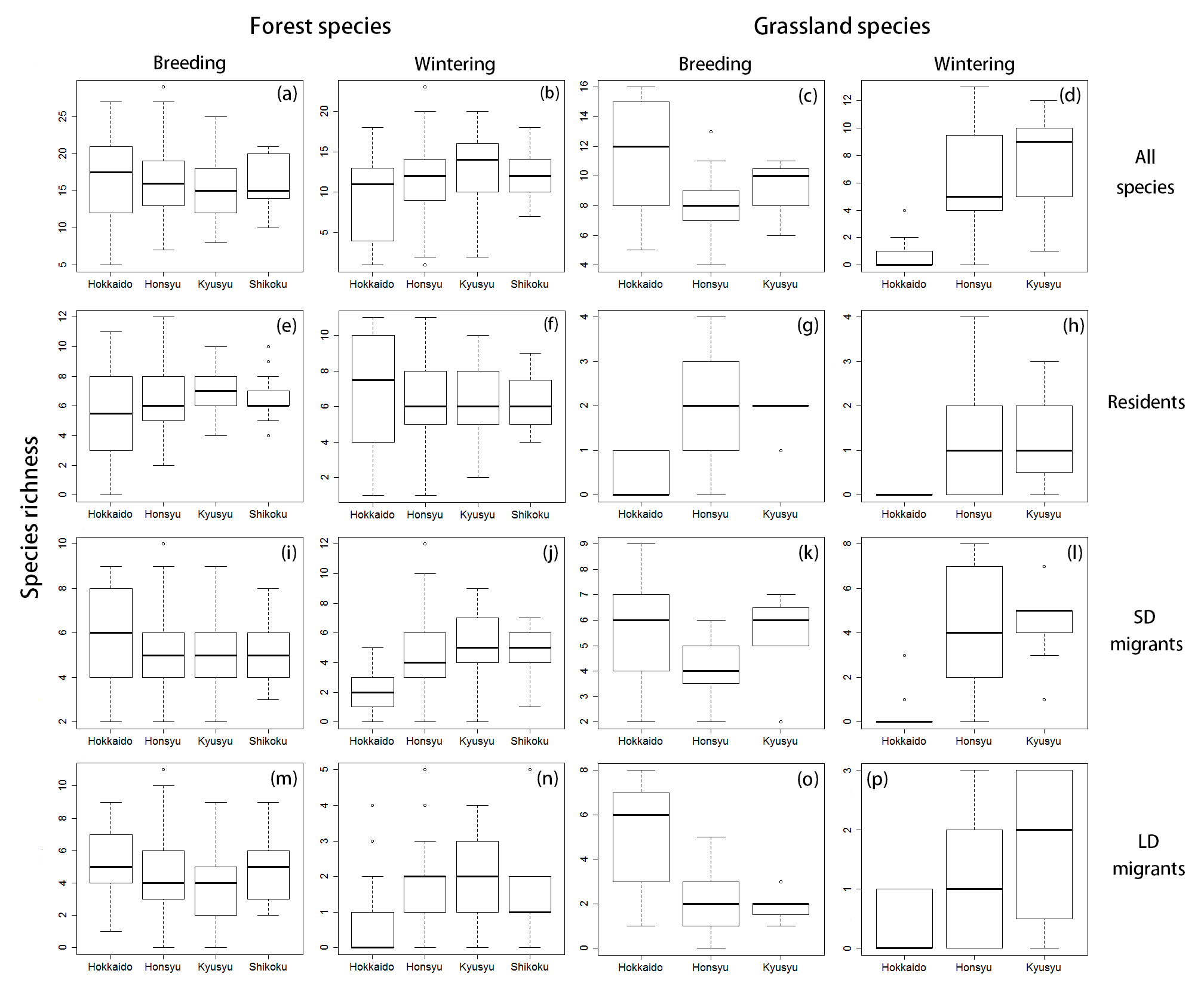


Appendix S1. Relationships between island size and species richness of forest birds [(a,b) all species, (e,f) residents, (i,j) short-distance migrants, (m,n) long-distance migrants] and grassland birds [(c,d) all species, (g,h) residents, (k,l) short-distance migrants, (o,p) long-distance migrants]. These results are from the breeding (a, c, e, g, i, k, m, o) and wintering (b, d, f, h, j, l, n, p) seasons. The size of the islands were as follows: Hokkaido: 77,984 km^2^; Honsyu: 227,942 km^2^; Kyusyu: 36,782 km^2^; Shikoku: 18,298 km^2^. Species richness did not increase on larger islands. Abbreviations: SD: short-distance, LD: long-distance, Breeding: breeding season, Wintering: wintering season.


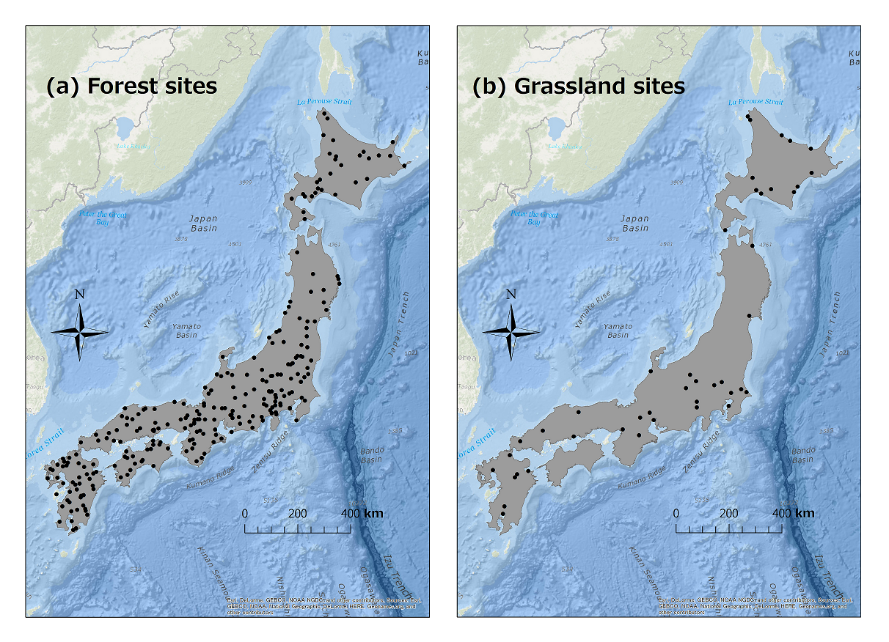


Appendix S2. The survey sites. The black dots indicate (a) 254 forest sites and (b) 43 grassland sites surveyed in the breeding and wintering seasons. Coordinates: 30°59′–45°31′N; 129°33′–145°49′E.

Appendix S3. Bird species subjected to analyses. “Season” indicates whether the species was detected in the breeding or the wintering season. For Siskin and Ashy minivet (only), we changed the migratory trait between seasons. We categorized 61 species as forest species (resident: 23, SD migrant: 17, LD migrant: 21) and 30 species as grassland species (resident: 5, SD migrant: 13, LD migrant: 12) in the breeding season, and 51 species as forest species (resident: 21, SD migrant: 17, LD migrant: 13) and 25 species as grassland species (resident: 5, SD migrant: 11, LD migrant: 9) in the wintering season. Abbreviations: Grass: grassland, SD: short-distance migrant, LD: long-distance migrant, B: breeding season, W: wintering season.

| Common name | Scientific name | Habitat | Migrant trait | Season |
| --- | --- | --- | --- | --- |
| Japanese Green Woodpecker | *Picus awokera* | Forest | Resident | B/W |
| Great Wpotted Woodpecker | *Dendrocopos major* | Forest | Resident | B/W |
| Hazel Grouse | *Tetrastes bonasia* | Forest | Resident | B |
| Long-tailed Tit | *Aegithalos caudatus* | Forest | Resident | B/W |
| White-backed Woodpecker | *Dendrocopos leucotos* | Forest | Resident | B/W |
| Azure-winged Magpie | *Cyanopica cyana* | Forest | Resident | B/W |
| Eurasian Jay | *Garrulus glandarius* | Forest | Resident | B/W |
| Brown Dipper | *Cinclus pallasii* | Forest | Resident | B/W |
| Treecreeper | *Certhia familiaris* | Forest | Resident | B/W |
| Black Woodpecker | *Dryocopus martius* | Forest | Resident | B/W |
| Lesser Spotted Woodpecker | *Dendrocopos minor* | Forest | Resident | B |
| Willow Tit | *Parus montanus* | Forest | Resident | B/W |
| Japanese Pygmy Woodpecker | *Dendrocopos kizuki* | Forest | Resident | B/W |
| Nuthatch | *Sitta europaea* | Forest | Resident | B/W |
| Great Tit | *Parus major* | Forest | Resident | B/W |
| Marsh Tit | *Parus palustris* | Forest | Resident | B/W |
| Jungle Crow | *Corvus macrorhynchos* | Forest | Resident | B/W |
| Carrion Crow | *Corvus corone* | Forest | Resident | B/W |
| Siskin | *Carduelis spinus* | Forest | Resident/LD | B/W |
| Varied Tit | *Parus varius* | Forest | Resident | B/W |
| Grey-headed Woodpecker | *Picus canus* | Forest | Resident | B/W |
| Greater Pied Kingfisher | *Ceryle lugubris* | Forest | Resident | B/W |
| Copper Pheasant | *Syrmaticus soemmerringii* | Forest | Resident | B/W |
| Japanese Green Pigeon | *Sphenurus sieboldii* | Forest | SD | B/W |
| Brown Thrush | *Turdus chrysolaus* | Forest | SD | B/W |
| Masked Grosbeak | *Eophona personata* | Forest | SD | B/W |
| Japanese Bush Warbler | *Cettia diphone* | Forest | SD | B/W |
| Eurasian Bullfinch | *Pyrrhula pyrrhula* | Forest | SD | B/W |
| Japanese Accentor | *Prunella rubida* | Forest | SD | W |
| Goldcrest | *Regulus regulus* | Forest | SD | B/W |
| Oriental Turtle Dove | *Streptopelia orientalis* | Forest | SD | B/W |
| Grey Wagtail | *Motacilla cinerea* | Forest | SD | B/W |
| Grey Bunting | *Emberiza variabilis* | Forest | SD | B/W |
| Hawfinch | *Coccothraustes coccothraustes* | Forest | SD | B/W |
| White's Thrush | *Zoothera dauma* | Forest | SD | B/W |
| Russet Sparrow | *Passer rutilans* | Forest | SD | B |
| Coal Tit | *Parus ater* | Forest | SD | B/W |
| Brown-eared Bulbul | *Hypsipetes amaurotis* | Forest | SD | B/W |
| Winter Wren | *Troglodytes troglodytes* | Forest | SD | B/W |
| Japanese White-eye | *Zosterops japonicus* | Forest | SD | B/W |
| Red-flanked Bushrobin | *Tarsiger cyanurus* | Forest | SD | B/W |
| Ruddy Kingfisher | *Halcyon coromanda* | Forest | LD | B |
| Eastern Pale-legged Leaf Warbler | *Phylloscopus borealoides* | Forest | LD | B |
| Blue-and-white Flycatcher | *Cyanoptila cyanomelana* | Forest | LD | B |
| Narcissus Flycatcher | *Ficedula narcissina* | Forest | LD | B |
| Grey Thrush | *Turdus cardis* | Forest | LD | B |
| Brown Flycatcher | *Muscicapa dauurica* | Forest | LD | B |
| Japanese Robin | *Erithacus akahige* | Forest | LD | B |
| Chestnut-cheeked Starling | *Sturnus philippensis* | Forest | LD | B |
| Siberian Blue Robin | *Luscinia cyane* | Forest | LD | B |
| Black Paradise Flycatcher | *Terpsiphone atrocaudata* | Forest | LD | B |
| Ashy Minivet | *Pericrocotus divaricatus* | Forest | LD/Resident | B/W |
| Horsfield's Hawk-cuckoo | *Cuculus fugax* | Forest | LD | B |
| Eastern Crowned Leaf Warbler | *Phylloscopus coronatus* | Forest | LD | B |
| Thick-billed Shrike | *Lanius tigrinus* | Forest | LD | B |
| Oriental Cuckoo | *Cuculus saturates* | Forest | LD | B |
| White-throated Needle-tailed Swift | *Hirundapus caudacutus* | Forest | LD | B |
| Little Cuckoo | *Cuculus poliocephalus* | Forest | LD | B |
| Siberian Thrush | *Turdus sibirica* | Forest | LD | B |
| Arctic Warbler | *Phylloscopus borealis* | Forest | LD | B |
| Fairy Pitta | *Pitta brachyuran* | Forest | LD | B |
| Short-tailed Bush Warbler | *Urosphena squameiceps* | Forest | LD | B |
| Brambling | *Fringilla montifringilla* | Forest | LD | W |
| Crossbill | *Loxia curvirostra* | Forest | LD | W |
| Pallas's Rosefinch | *Carpoducus roseus* | Forest | LD | W |
| Red-breasted Flycatcher | *Ficedula parva* | Forest | LD | W |
| Waxwing | *Bombycilla garrulus* | Forest | LD | W |
| Pine Grosbeak | *Pinicola enucleator* | Forest | LD | W |
| Pale Thrush | *Turdus pallidus* | Forest | LD | W |
| Dusky Thrush | *Turdus naumanni* | Forest | LD | W |
| Japanese Waxwing | *Bombycilla japonica* | Forest | LD | W |
| Eye-browed Thrush | *Turdus obscurus* | Forest | LD | W |
| Yellow-throated Bunting | *Emberiza elegans* | Forest | LD | W |
| Raven | *Corvus corax* | Forest | LD | W |
| Common Pheasant | *Phasianus colchicus* | Grass | Resident | B/W |
| Japanese Reed Bunting | *Emberiza yessoensis* | Grass | Resident | B/W |
| Eurasian Tree Sparrow | *Passer montanus* | Grass | Resident | B/W |
| Japanese Wagtail | *Motacilla grandis* | Grass | Resident | B/W |
| Grey Starling | *Sturnus cineraceus* | Grass | Resident | B/W |
| Black-faced Bunting | *Emberiza spodocephala* | Grass | SD | B/W |
| Wryneck | *Jynx torquilla* | Grass | SD | B |
| Reed Bunting | *Emberiza schoeniclus* | Grass | SD | B/W |
| Japanese Marsh Warbler | *Locustella pryeri* | Grass | SD | B |
| Oriental Greenfinch | *Carduelis sinica minor* | Grass | SD | B/W |
| Fan-tailed Warbler | *Cisticola juncidis* | Grass | SD | B/W |
| White Wagtail | *Motacilla lugens* | Grass | SD | B/W |
| Common Skylark | *Alauda arvensis* | Grass | SD | B/W |
| Olive-backed Pipit | *Anthus hodgsoni* | Grass | SD | B/W |
| Long-tailed Rosefinch | *Uragus sibiricus* | Grass | SD | B/W |
| Chestnut-eared Bunting | *Emberiza fucata* | Grass | SD | B/W |
| Siberian Meadow Bunting | *Emberiza cioides* | Grass | SD | B/W |
| Bull-headed Shrike | *Lanius bucephalus* | Grass | SD | B/W |
| Grey's Grasshopper Warbler | *Locustella fasciolata* | Grass | LD | B |
| Latham's Snipe | *Gallinago hardwickii* | Grass | LD | B |
| Great Reed Warbler | *Acrocephalus arundinaceus* | Grass | LD | B |
| Common Cuckoo | *Cuculus canorus* | Grass | LD | B |
| Black-browed Reed Warbler | *Acrocephalus bistrigiceps* | Grass | LD | B |
| Middendorff's Grasshopper Warbler | *Locustella ochotensis* | Grass | LD | B |
| Sand Martin | *Riparia riparia* | Grass | LD | B |
| House Swallow | *Hirundo rustica* | Grass | LD | B |
| Yellow Wagtail | *Motacilla flava* | Grass | LD | B |
| Siberian Rubythroat | *Luscinia calliope* | Grass | LD | B |
| Stonechat | *Saxicola torquata* | Grass | LD | B |
| Lanceolated Grasshopper Warbler | *Locustella lanceolate* | Grass | LD | B |
| Great Grey Shrike | *Lanius excubitor* | Grass | LD | W |
| Rustic Bunting | *Emberiza rustica* | Grass | LD | W |
| Daurian Redstart | *Phoenicurus auroreus* | Grass | LD | W |
| Water Pipit | *Anthus spinoletta* | Grass | LD | W |
| Lapland Bunting | *Calcarius lapponicus* | Grass | LD | W |
| European Penduline Tit | *Remiz pendulinus* | Grass | LD | W |
| Rosy Finch | *Leucosticte arctoa* | Grass | LD | W |
| Redpoll | *Carduelis flammea* | Grass | LD | W |
| Rook | *Corvus frugilegus* | Grass | LD | W |


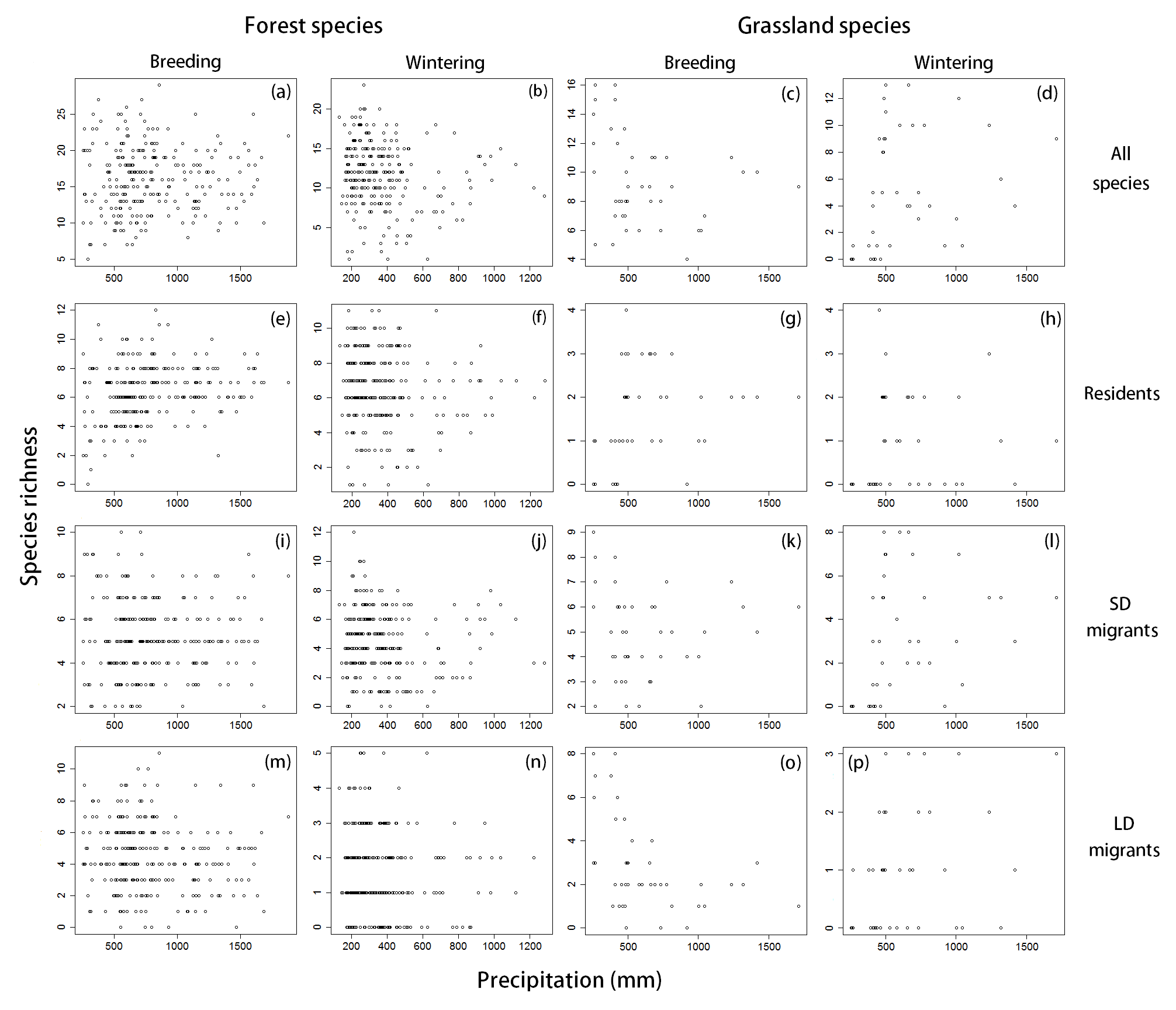


Appendix S4. Relationships between seasonal precipitation and species richness of forest birds [(a,b) all species, (e,f) residents, (i,j) short-distance migrants, (m,n) long-distance migrants] and grassland birds [(c,d) all species, (g,h) residents, (k,l) short-distance migrants, (o,p) long-distance migrants]. These results are from the breeding (a, c, e, g, i, k, m, o) and wintering (b, d, f, h, j, l, n, p) seasons. There are no clear relationships. Abbreviations: SD: short-distance, LD: long-distance, Breeding: breeding season, Wintering: wintering season.

Appendix S5. Comparison of the effects of annual mean temperature and mean temperature during each season (estimates ± standard errors [Wald *p*-value], and ΔAkaike information criterion [AIC]). We constructed generalized linear mixed models (GLMMs) with random site effects. In each model, we considered one explanatory variable (i.e., annual mean temperature, mean temperature during the breeding season, or mean temperature during the wintering season) and its squared term. Each row of the table shows the results of one model. Abbreviations: Breeding: breeding season; Wintering: wintering season; TEMP: mean temperature during each season.

| All forest species | | | | | | | | |
| --- | --- | --- | --- | --- | --- | --- | --- | --- |
| Season |  | Breeding | | |  | Wintering | | |
|  |  | TEMP | (TEMP)^2^ | ΔAIC |  | TEMP | (TEMP)^2^ | ΔAIC |
| Annual |  | -0.10±0.02 (<0.01) | -0.07±0.02 (<0.01) | 0 |  | 0.06±0.02 (<0.01) | -0.07±0.02 (<0.01) | 0 |
| Sumer |  | -0.09±0.02 (<0.01) | -0.06±0.02 (<0.01) | 6.5 |  | 0.06±0.02 (0.01) | -0.06±0.02 (<0.01) | 2.3 |
| Winter |  | -0.10±0.02 (<0.01) | -0.06±0.01 (<0.01) | 3.6 |  | 0.07±0.02 (<0.01) | -0.05±0.02 (<0.01) | 2.8 |

| Forest residents | | | | | | | | |
| --- | --- | --- | --- | --- | --- | --- | --- | --- |
| Season |  | Breeding | | |  | Wintering | | |
|  |  | TEMP | (TEMP)^2^ | ΔAIC |  | TEMP | (TEMP)^2^ | ΔAIC |
| Annual |  | -0.03±0.03 (0.35) | -0.07±0.02 (<0.01) | 0 |  | -0.06±0.03 (0.02) | -0.05±0.02 (0.03) | 0 |
| Sumer |  | -0.02±0.03 (0.41) | -0.06±0.02 (0.01) | 3.2 |  | -0.06±0.03 (0.02) | -0.04±0.02 (0.06) | 0.7 |
| Winter |  | -0.02±0.03 (0.38) | -0.06±0.02 (<0.01) | 0.2 |  | -0.06±0.03 (0.03) | -0.04±0.02 (0.05) | 1.2 |

| Forest short-distance migrants | | | | | | | | |
| --- | --- | --- | --- | --- | --- | --- | --- | --- |
| Season |  | Breeding | | |  | Wintering | | |
|  |  | TEMP | (TEMP)^2^ | ΔAIC |  | TEMP | (TEMP)^2^ | ΔAIC |
| Annual |  | -0.10±0.03 (<0.01) | -0.05±0.03 (0.05) | 0 |  | 0.24±0.03 (<0.01) | -0.11±0.03 (<0.01) | 0.6 |
| Sumer |  | -0.09±0.03 (<0.01) | -0.03±0.03 (0.20) | 1.4 |  | 0.23±0.03 (<0.01) | -0.12±0.03 (<0.01) | 0 |
| Winter |  | -0.10±0.03 (<0.01) | -0.04±0.02 (0.06) | 0.9 |  | 0.24±0.03 (<0.01) | -0.08±0.03 (<0.01) | 5.7 |

Appendix S5, continued.

| Forest long-distance migrants | | | | | | | | |
| --- | --- | --- | --- | --- | --- | --- | --- | --- |
| Season |  | Breeding | | |  | Wintering | | |
|  |  | TEMP | (TEMP)^2^ | ΔAIC |  | TEMP | (TEMP)^2^ | ΔAIC |
| Annual |  | -0.20±0.04 (<0.01) | -0.10±0.03 (<0.01) | 0 |  | 0.18±0.06 (<0.01) | -0.13±0.05 (0.01) | 1.0 |
| Sumer |  | -0.20±0.04 (<0.01) | -0.09±0.03 (<0.01) | 1.6 |  | 0.17±0.06 (<0.01) | -0.13±0.05 (0.02) | 2.5 |
| Winter |  | -0.20±0.04 (<0.01) | -0.09±0.02 (<0.01) | 3.0 |  | 0.19±0.06 (<0.01) | -0.12±0.05 (0.02) | 0 |

| All grassland species | | | | | | | | |
| --- | --- | --- | --- | --- | --- | --- | --- | --- |
| Season |  | Breeding | | |  | Wintering | | |
|  |  | TEMP | (TEMP)^2^ | ΔAIC |  | TEMP | (TEMP)^2^ | ΔAIC |
| Annual |  | -0.12±0.05 (0.02) | 0.14±0.07 (0.05) | 0.5 |  | 1.00±0.12 (<0.01) | -0.36±0.13 (<0.01) | 2.5 |
| Sumer |  | -0.11±0.05 (0.02) | 0.14±0.07 (0.05) | 0.4 |  | 1.02±0.13 (<0.01) | -0.37±0.13 (<0.01) | 0 |
| Winter |  | -0.11±0.05 (0.02) | 0.14±0.07 (0.05) | 0 |  | 0.98±0.12 (<0.01) | -0.29±0.13 (0.02) | 5.3 |

| Grassland residents | | | | | | | | |
| --- | --- | --- | --- | --- | --- | --- | --- | --- |
| Season |  | Breeding | | |  | Wintering | | |
|  |  | TEMP | (TEMP)^2^ | ΔAIC |  | TEMP | (TEMP)^2^ | ΔAIC |
| Annual |  | 0.67±0.17 (<0.01) | -0.28±0.19 (0.15) | 2.2 |  | 1.51±0.43 (<0.01) | -0.52±0.34 (0.12) | 1.8 |
| Sumer |  | 0.72±0.18 (<0.01) | -0.31±0.20 (0.12) | 0 |  | 1.64±0.49 (<0.01) | -0.59±0.38 (0.12) | 0 |
| Winter |  | 0.64±0.17 (<0.01) | -0.25±0.18 (0.17) | 3.5 |  | 1.50±0.44 (<0.01) | -0.52±0.34 (0.12) | 3.1 |

Appendix S5, continued.

| Grassland short-distance migrants | | | | | | | | |
| --- | --- | --- | --- | --- | --- | --- | --- | --- |
| Season |  | Breeding | | |  | Wintering | | |
|  |  | TEMP | (TEMP)^2^ | ΔAIC |  | TEMP | (TEMP)^2^ | ΔAIC |
| Annual |  | -0.12±0.07 (0.07) | 0.12±0.10 (0.23) | 0.1 |  | 0.98±0.15 (<0.01) | -0.41±0.15 (<0.01) | 0.9 |
| Sumer |  | -0.12±0.07 (0.08) | 0.10±0.10 (0.32) | 0.7 |  | 1.00±0.15 (<0.01) | -0.44±0.15 (<0.01) | 0 |
| Winter |  | -0.11±0.07 (0.11) | 0.13±0.10 (0.19) | 0 |  | 0.97±0.15 (<0.01) | -0.34±0.14 (0.02) | 1.4 |

| Grassland long distance migrants | | | | | | | | |
| --- | --- | --- | --- | --- | --- | --- | --- | --- |
| Season |  | Breeding | | |  | Wintering | | |
|  |  | TEMP | (TEMP)^2^ | ΔAIC |  | TEMP | (TEMP)^2^ | ΔAIC |
| Annual |  | -0.42±0.09 (<0.01) | 0.24±0.14 (0.08) | 2.6 |  | 0.79±0.22 (<0.01) | -0.19±0.24 (0.42) | 1.0 |
| Sumer |  | -0.42±0.10 (<0.01) | 0.24±0.13 (0.07) | 0 |  | 0.81±0.22 (<0.01) | -0.14±0.25 (0.56) | 0 |
| Winter |  | -0.42±0.10 (<0.01) | 0.20±0.13 (0.13) | 2.3 |  | 0.74±0.21 (<0.01) | -0.15±0.23 (0.51) | 2.7 |


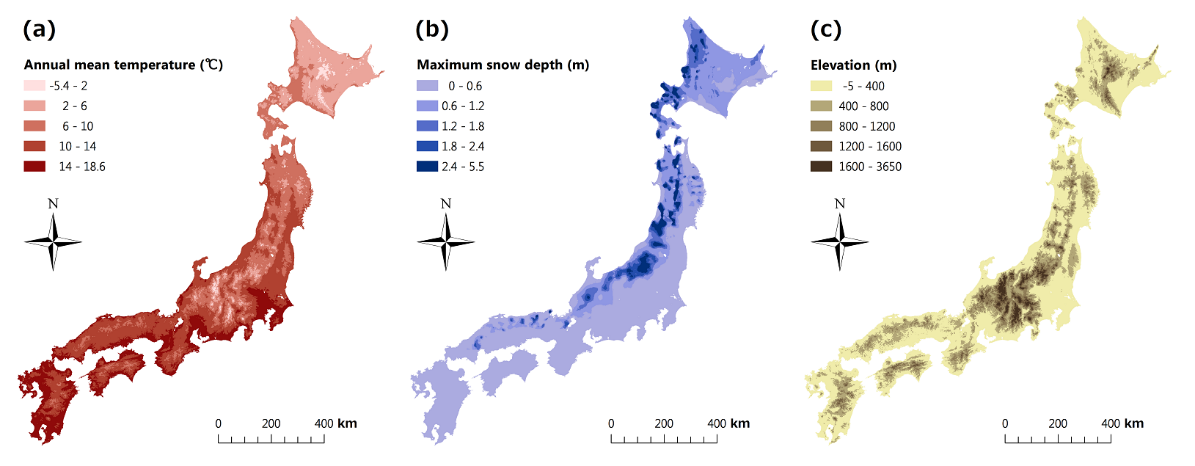


Appendix S6. (a) The annual mean temperature, (b) The maximum snow depth, and (c) The elevation at each grid (1 km^2^).


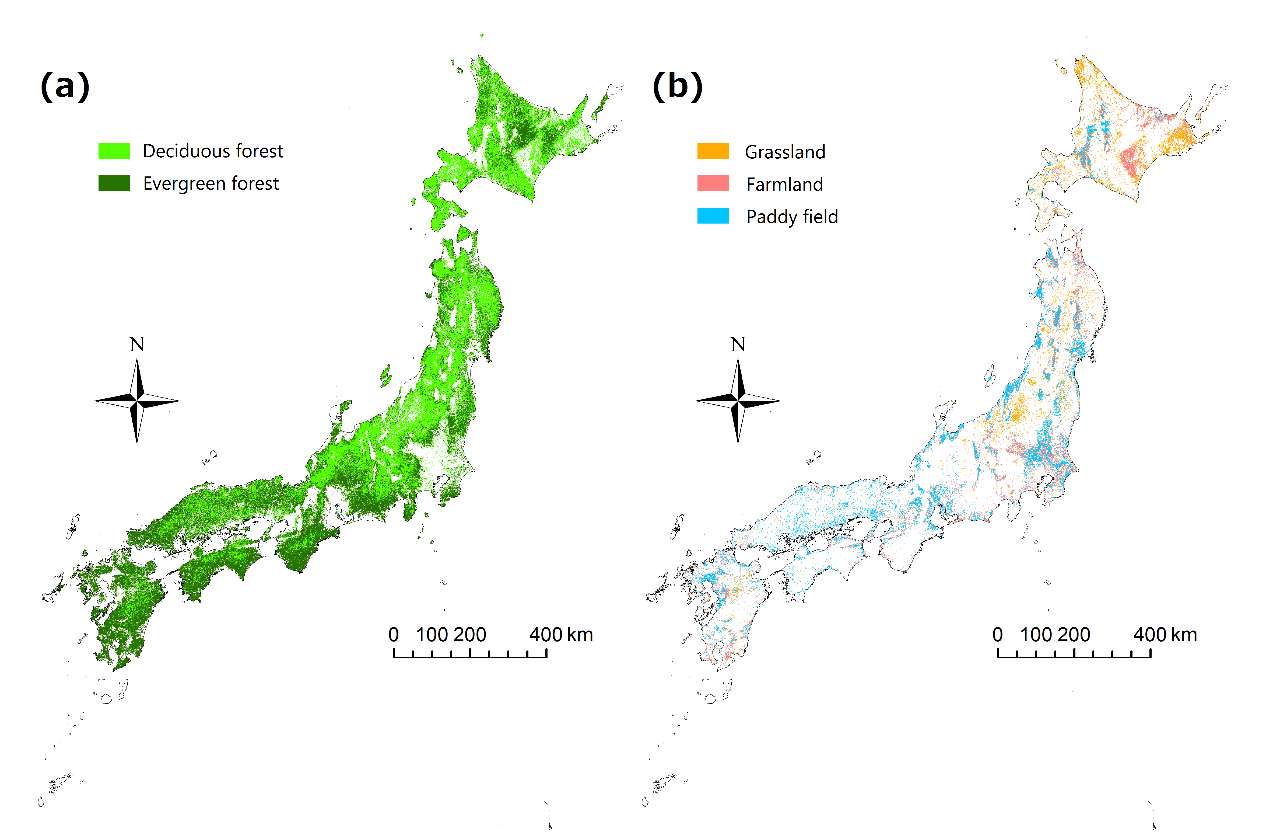


Appendix S7. Distributions of (a) evergreen and deciduous forests and (b) grasslands, farmlands, and paddy fields.

Appendix S8. Comparison of the effects of the extent of surrounding habitat within each scale (estimates ± standard errors [Wald *p*-value], and ΔAIC). We constructed GLMMs with random site effects. In each model, we considered one explanatory variable (i.e., the extent of surrounding habitat within 1.25, 2.5, 5, 10, or 15 km) and its squared term. Each row of the table shows the results of one model. Abbreviations: Breeding: breeding season; Wintering: wintering season; AREA: the extent of surrounding habitat within each scale.

| All forest species | | | | | | | |
| --- | --- | --- | --- | --- | --- | --- | --- |
| Scale | Breeding | | |  | Wintering | | |
|  | AREA | (AREA)^2^ | ΔAIC |  | AREA | (AREA)^2^ | ΔAIC |
| 1.25 | 0.07±0.03 (0.01) | -0.01±0.02 (0.44) | 0 |  | 0.04±0.04 (0.23) | 0.01±0.02 (0.51) | 3.3 |
| 2.5 | 0.07±0.03 (<0.01) | -0.01±0.02 (0.59) | 2.9 |  | 0.03±0.04 (0.44) | 0.01±0.02 (0.55) | 4.4 |
| 5 | 0.06±0.02 (0.02) | -0.02±0.02 (0.34) | 6.3 |  | -0.04±0.03 (0.23) | -0.02±0.02 (0.40) | 3.6 |
| 10 | 0.05±0.02 (0.02) | -0.02±0.02 (0.30) | 11.6 |  | -0.06±0.03 (0.03) | -0.03±0.02 (0.17) | 0.1 |
| 15 | 0.05±0.02 (<0.01) | -0.03±0.02 (0.11) | 11.8 |  | -0.05±0.02 (0.03) | -0.03±0.02 (0.18) | 0 |

| Forest residents | | | | | | | |
| --- | --- | --- | --- | --- | --- | --- | --- |
| Scale | Breeding | | |  | Wintering | | |
|  | AREA | (AREA)^2^ | ΔAIC |  | AREA | (AREA)^2^ | ΔAIC |
| 1.25 | 0.10±0.04 (0.02) | 0.02±0.02 (0.54) | 0 |  | 0.04±0.04 (0.32) | 0.01±0.02 (0.71) | 0.2 |
| 2.5 | 0.10±0.04 (0.02) | 0.03±0.03 (0.34) | 1.5 |  | 0.05±0.04 (0.23) | 0.02±0.03 (0.51) | 0 |
| 5 | 0.08±0.04 (0.04) | 0.02±0.03 (0.52) | 3.2 |  | 0.01±0.04 (0.82) | -0.00±0.03 (0.87) | 1.4 |
| 10 | 0.06±0.03 (0.06) | 0.01±0.03 (0.82) | 4.4 |  | -0.01±0.03 (0.76) | -0.02±0.03 (0.53) | 1.3 |
| 15 | 0.06±0.03 (0.04) | -0.00±0.02 (0.98) | 4.2 |  | -0.01±0.03 (0.64) | -0.03±0.02 (0.27) | 0.4 |

Appendix S8, continued.

| Forest short-distance migrants | | | | | | | |
| --- | --- | --- | --- | --- | --- | --- | --- |
| Scale | Breeding | | |  | Wintering | | |
|  | AREA | (AREA)^2^ | ΔAIC |  | AREA | (AREA)^2^ | ΔAIC |
| 1.25 | 0.03±0.05 (0.53) | -0.04±0.03 (0.18) | 0 |  | 0.02±0.05 (0.71) | -0.01±0.03 (0.83) | 11.4 |
| 2.5 | 0.05±0.04 (0.31) | -0.03±0.03 (0.35) | 1.1 |  | -0.03±0.05 (0.57) | -0.02±0.04 (0.56) | 11.9 |
| 5 | 0.04±0.04 (0.32) | -0.03±0.03 (0.29) | 1.9 |  | -0.11±0.04 (0.01) | -0.06±0.03 (0.07) | 5.9 |
| 10 | 0.03±0.03 (0.45) | -0.04±0.03 (0.13) | 2.9 |  | -0.14±0.04 (<0.01) | -0.06±0.03 (0.04) | 0 |
| 15 | 0.02±0.03 (0.43) | -0.05±0.03 (0.04) | 2.0 |  | -0.11±0.03 (<0.01) | -0.04±0.03 (0.13) | 2.4 |

| Forest long-distance migrants | | | | | | | |
| --- | --- | --- | --- | --- | --- | --- | --- |
| Scale | Breeding | | |  | Wintering | | |
|  | AREA | (AREA)^2^ | ΔAIC |  | AREA | (AREA)^2^ | ΔAIC |
| 1.25 | 0.09±0.05 (0.09) | -0.03±0.03 (0.40) | 0.2 |  | 0.06±0.08 (0.43) | 0.07±0.04 (0.11) | 0.5 |
| 2.5 | 0.07±0.05 (0.14) | -0.05±0.04 (0.21) | 0 |  | 0.06±0.08 (0.45) | 0.09±0.05 (0.10) | 0 |
| 5 | 0.05±0.04 (0.23) | -0.06±0.03 (0.10) | 1.6 |  | -0.05±0.07 (0.52) | 0.03±0.05 (0.62) | 1 |
| 10 | 0.06±0.04 (0.09) | -0.03±0.03 (0.42) | 7.5 |  | -0.10±0.06 (0.13) | -0.01±0.05 (0.85) | 0.4 |
| 15 | 0.06±0.03 (0.06) | -0.03±0.03 (0.31) | 8.2 |  | -0.09±0.05 (0.12) | 0.00±0.04 (0.94) | 0.4 |

| All grassland species | | | | | | | |
| --- | --- | --- | --- | --- | --- | --- | --- |
| Scale | Breeding | | |  | Wintering | | |
|  | AREA | (AREA)^2^ | ΔAIC |  | AREA | (AREA)^2^ | ΔAIC |
| 1.25 | 0.06±0.05 (0.25) | -0.08±0.06 (0.18) | 0.7 |  | 0.27±0.18 (0.13) | -0.15±0.19 (0.42) | 2.4 |
| 2.5 | 0.07±0.05 (0.21) | -0.09±0.05 (0.10) | 0.1 |  | 0.37±0.17 (0.03) | -0.24±0.18 (0.18) | 0 |
| 5 | 0.11±0.06 (0.07) | -0.08±0.05 (0.15) | 0 |  | 0.40±0.19 (0.04) | -0.21±0.18 (0.23) | 1.1 |
| 10 | 0.11±0.06 (0.10) | -0.05±0.05 (0.36) | 1.0 |  | 0.34±0.21 (0.10) | -0.10±0.18 (0.58) | 2.5 |
| 15 | 0.09±0.06 (0.15) | -0.03±0.05 (0.52) | 1.6 |  | 0.33±0.21 (0.12) | -0.05±0.16 (0.77) | 2.4 |

Appendix S8, continued.

| Grassland residents | | | | | | | |
| --- | --- | --- | --- | --- | --- | --- | --- |
| Scale | Breeding | | |  | Wintering | | |
|  | AREA | (AREA)^2^ | ΔAIC |  | AREA | (AREA)^2^ | ΔAIC |
| 1.25 | 0.24±0.14 (0.09) | -0.16±0.15 (0.27) | 0.5 |  | 0.19±0.21 (0.37) | -0.18±0.23 (0.46) | 2 |
| 2.5 | 0.29±0.14 (0.04) | -0.08±0.13 (0.53) | 0 |  | 0.37±0.23 (0.10) | -0.26±0.22 (0.25) | 0 |
| 5 | 0.27±0.15 (0.08) | -0.09±0.13 (0.48) | 1.2 |  | 0.41±0.24 (0.10) | -0.28±0.22 (0.20) | 0.2 |
| 10 | 0.25±0.16 (0.12) | -0.09±0.13 (0.50) | 1.9 |  | 0.33±0.26 (0.20) | -0.15±0.21 (0.48) | 1.7 |
| 15 | 0.24±0.16 (0.13) | -0.05±0.11 (0.66) | 1.7 |  | 0.33±0.26 (0.20) | -0.10±0.19 (0.60) | 1.6 |

| Grassland short-distance migrants | | | | | | | |
| --- | --- | --- | --- | --- | --- | --- | --- |
| Scale | Breeding | | |  | Wintering | | |
|  | AREA | (AREA)^2^ | ΔAIC |  | AREA | (AREA)^2^ | ΔAIC |
| 1.25 | -0.03±0.07 (0.63) | -0.07±0.08 (0.37) | 2.1 |  | 0.21±0.16 (0.20) | -0.11±0.17 (0.52) | 2.1 |
| 2.5 | -0.05±0.07 (0.47) | -0.11±0.08 (0.14) | 0 |  | 0.29±0.16 (0.07) | -0.22±0.17 (0.19) | 0 |
| 5 | -0.05±0.08 (0.51) | -0.03±0.07 (0.73) | 2.3 |  | 0.33±0.18 (0.07) | -0.20±0.17 (0.24) | 0.8 |
| 10 | -0.06±0.08 (0.48) | 0.03±0.07 (0.70) | 2.6 |  | 0.31±0.19 (0.11) | -0.10±0.16 (0.55) | 1.5 |
| 15 | -0.06±0.09 (0.47) | 0.03±0.07 (0.61) | 2.6 |  | 0.32±0.19 (0.10) | -0.07±0.15 (0.62) | 1.1 |

| Grassland long-distance migrants | | | | | | | |
| --- | --- | --- | --- | --- | --- | --- | --- |
| Scale | Breeding | | |  | Wintering | | |
|  | AREA | (AREA)^2^ | ΔAIC |  | AREA | (AREA)^2^ | ΔAIC |
| 1.25 | 0.13±0.12 (0.27) | -0.06±0.13 (0.64) | 4.3 |  | 0.33±0.18 (0.08) | -0.23±0.20 (0.24) | 2.8 |
| 2.5 | 0.16±0.12 (0.20) | -0.08±0.12 (0.49) | 3.9 |  | 0.46±0.20 (0.02) | -0.22±0.18 (0.23) | 0.5 |
| 5 | 0.32±0.14 (0.02) | -0.19±0.12 (0.11) | 0 |  | 0.50±0.21 (0.02) | -0.14±0.17 (0.40) | 0 |
| 10 | 0.34±0.15 (0.02) | -0.17±0.12 (0.13) | 0.5 |  | 0.44±0.21 (0.04) | -0.07±0.15 (0.63) | 0.8 |
| 15 | 0.30±0.15 (0.05) | -0.15±0.11 (0.17) | 1.7 |  | 0.38±0.21 (0.07) | -0.03±0.13 (0.83) | 1.5 |


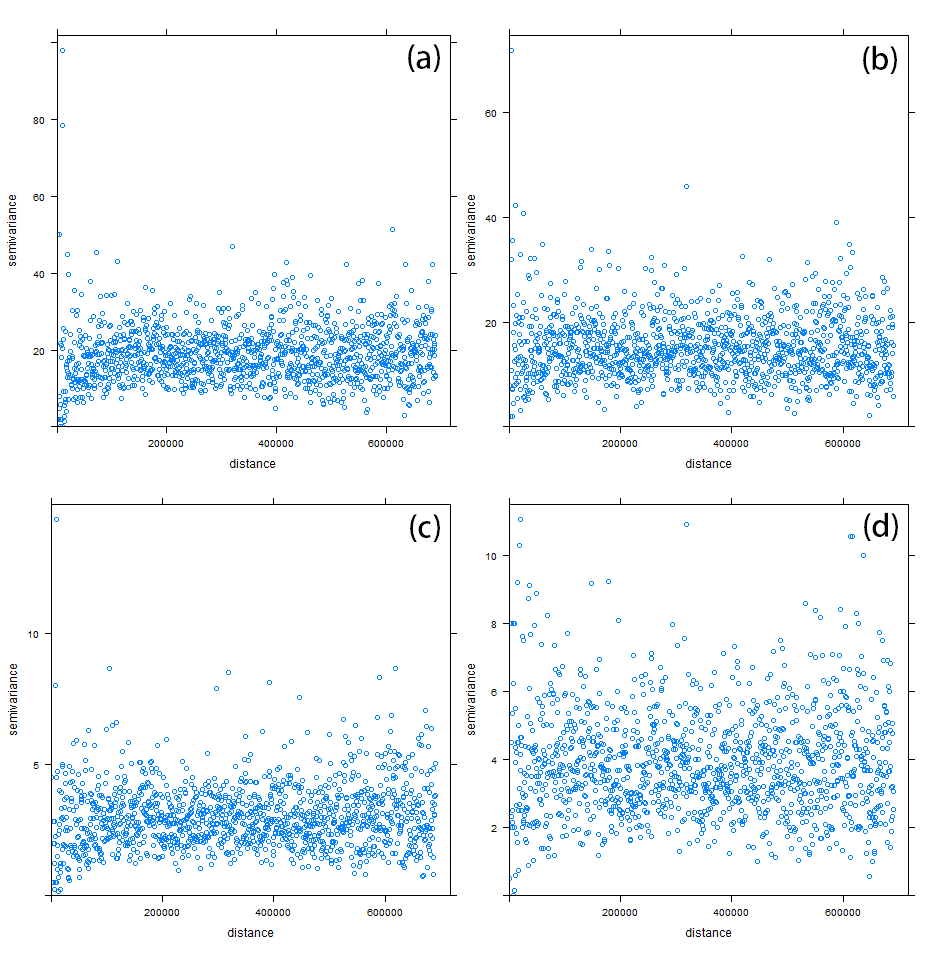


Appendix S9. Semi-variogram for species richness of forest birds [(a,b) all species, (c,d) residents, (e,f) short-distance migrants, (g,h) long-distance migrants] and grassland birds [(i,j) all species, (k,l) residents, (m,n) short-distance migrants, (o,p) long-distance migrants]. These results are from the breeding (a, c, e, g, i, k, m, o) and wintering (b, d, f, h, j, l, n, p) seasons.


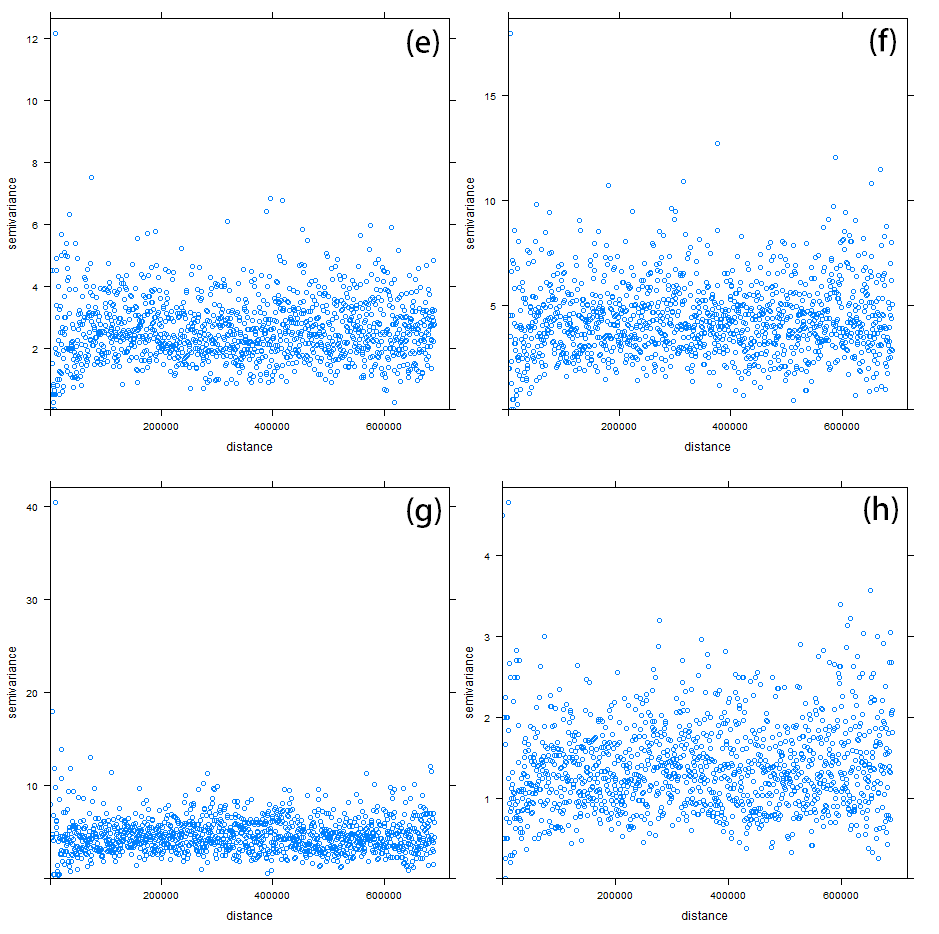


Appendix S9, continued.


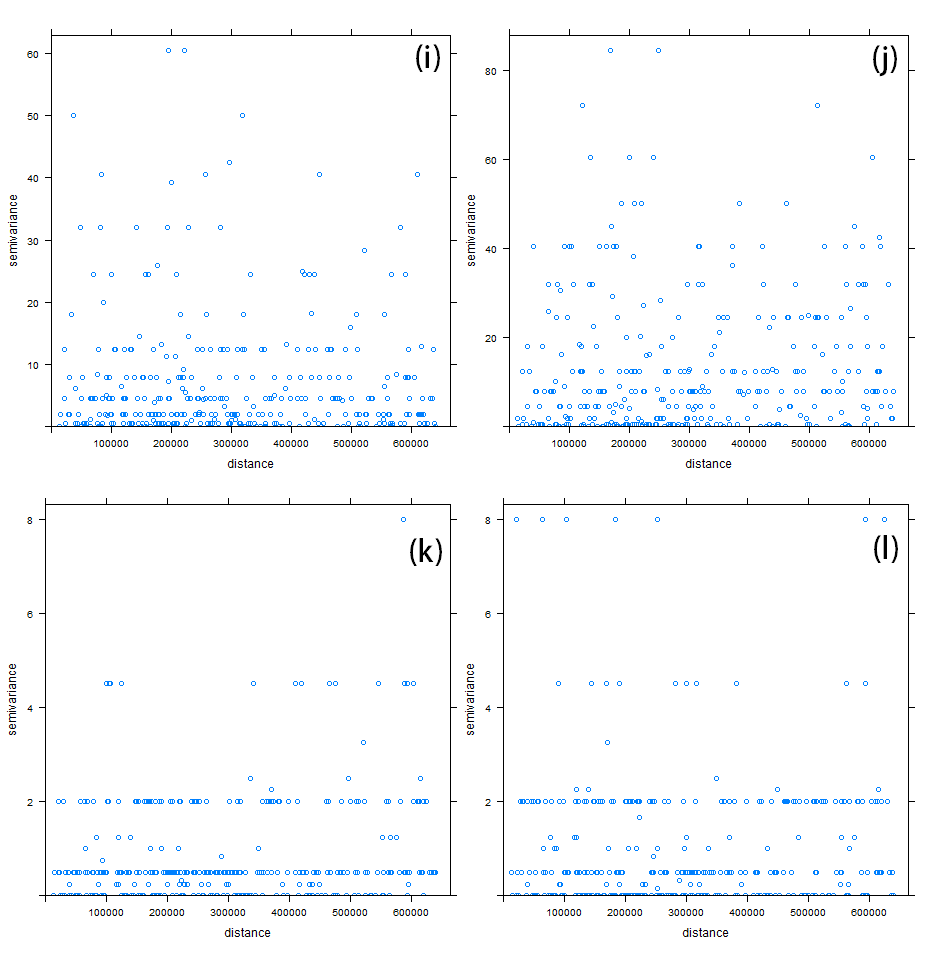


Appendix S9, continued.


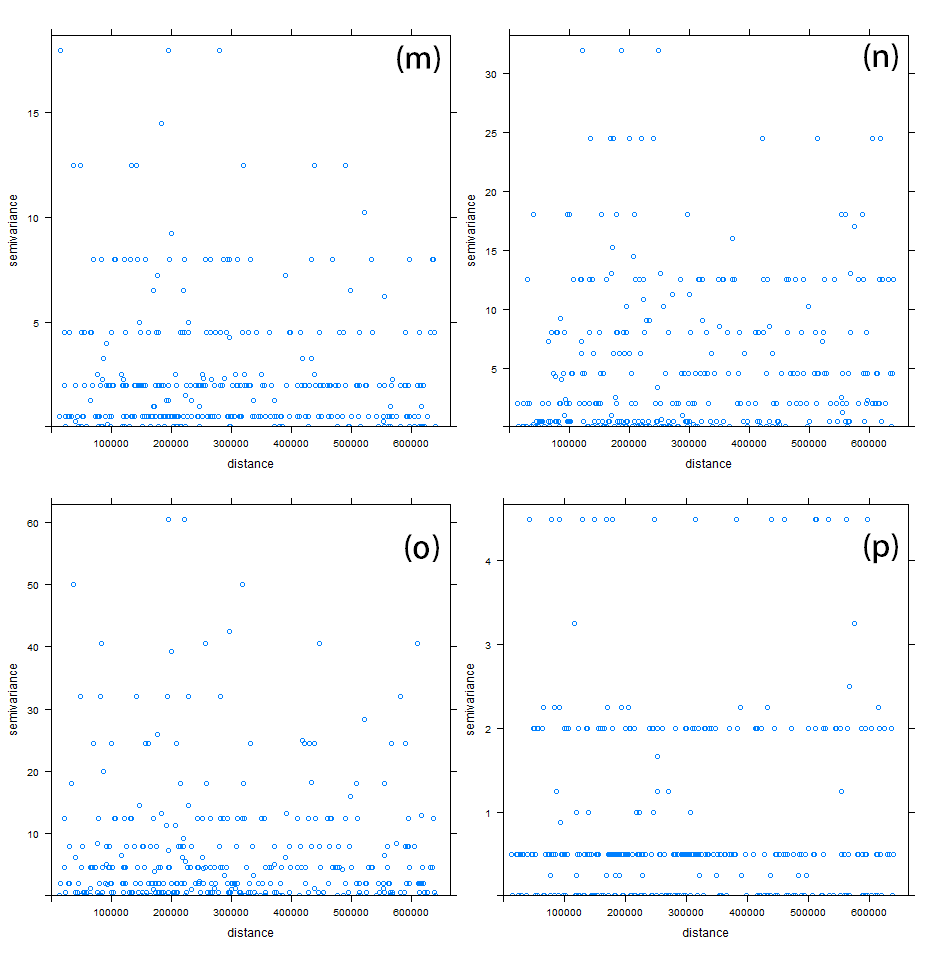


Appendix S9, continued.

Appendix S10. Variance inflation factors using linear values for each environmental factor in the analyses of all species in each habitat and season. Abbreviations: Breeding: breeding season; Wintering: wintering season; TEMP: annual mean temperature; SNOW: maximum snow depth; ELEV: elevation; AREA: area of surrounding suitable habitat (within a radius of 1.25 km for breeding forest birds, 10 km for wintering forest birds, and 5 km for grassland birds in both seasons). Cross marks: explanatory variables that were not used in construction of the full model to avoid multicollinearity.

| Variables | Forest | |  | Grassland | |
| --- | --- | --- | --- | --- | --- |
|  | Breeding | Wintering |  | Breeding | Wintering |
| TEMP | 1.28 | 2.69 |  | 1.05 | 1.38 |
| SNOW | × | 1.99 |  | × | × |
| ELEV | 1.65 | 2.30 |  | 1.25 | 1.53 |
| AREA | 1.34 | 1.62 |  | 1.21 | 1.12 |


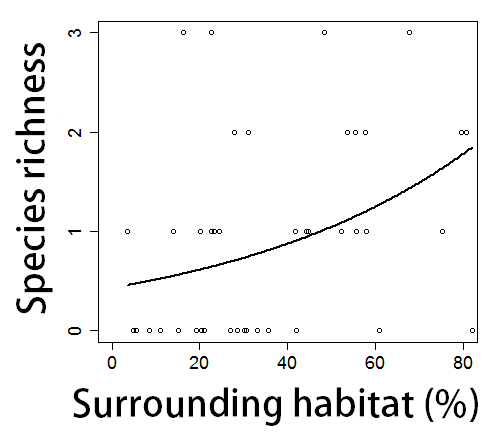


Appendix S11. Relationship between the extent of surrounding open habitat within 5 km and the species richness of grassland long-distance migrants, in the wintering season. Dots: Data collected at each site; solid lines: estimates afforded by the best spatial models. We used a GLMM (a multivariate analysis), and the effects of the other explanatory variables were considered and fixed to mean values in the figure. See details of the best spatial model in Table 1.


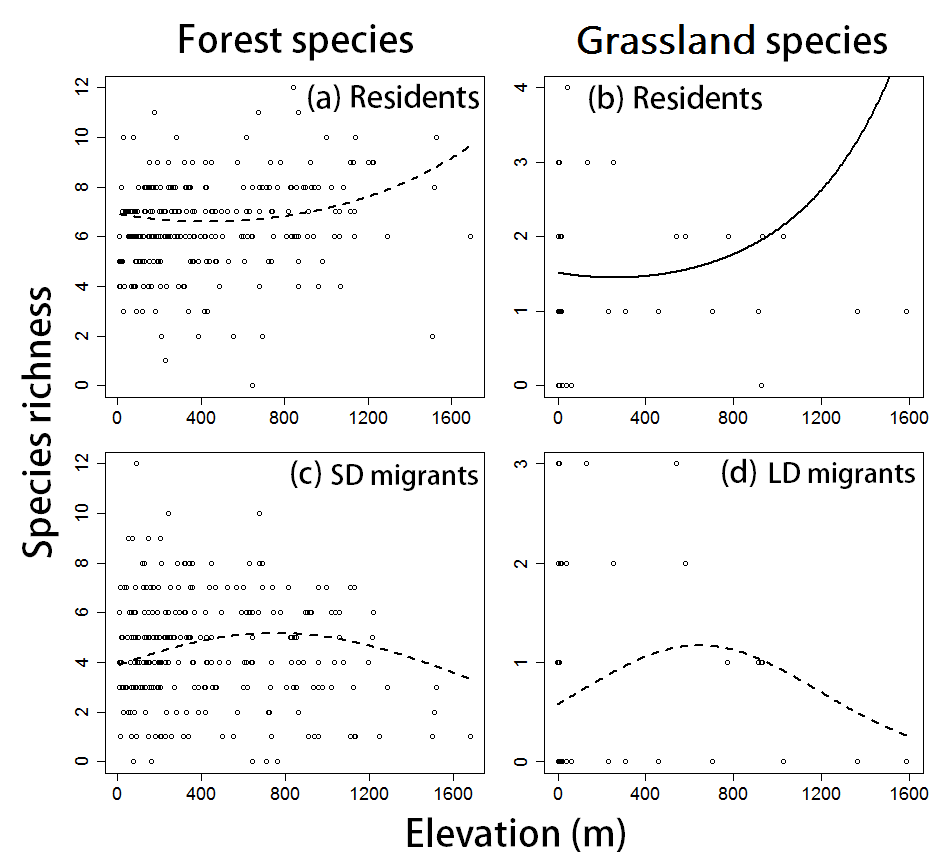


Appendix S12. Relationship between elevation and species richness of forest species: [(a) residents in the breeding season, (c) short distance migrants in the wintering season] and grassland species: [(b) residents in the breeding season, (d) long-distance migrants in the wintering season]. Dots: data derived at each site; lines: estimates afforded by the best spatial models (solid lines indicate semi-partial R^2^ of either the temperature and its squared term > 0.1, and broken lines indicate that of both the temperature and its squared term < 0.1). We used a GLMM (a multivariate analysis), and the effects of the other explanatory variables were considered and fixed to mean values in each figure. See details of the best spatial models in Table 1. Abbreviations: LD: long distance, SD: short distance.


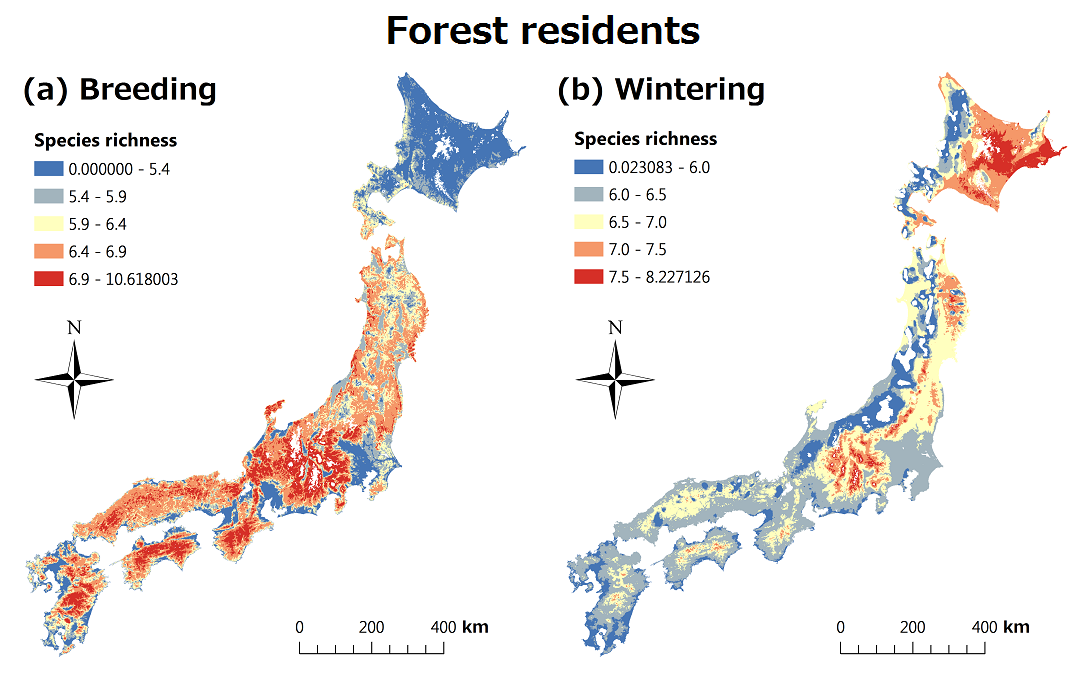


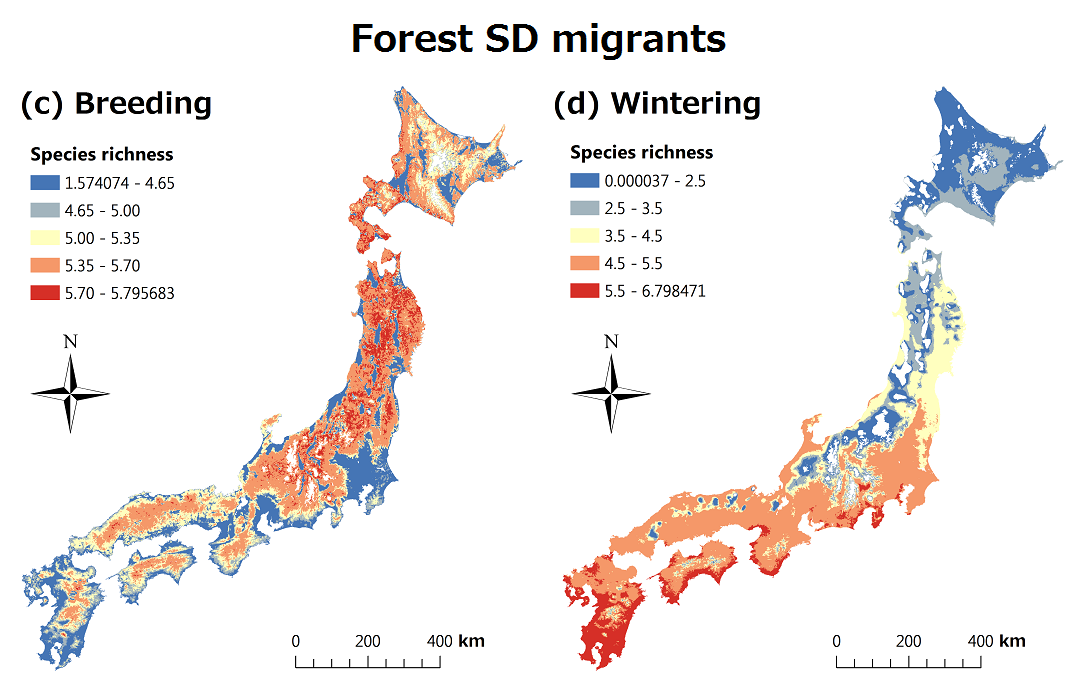


Appendix S13. Expected species richness of forest (a,b) residents, (c,d) short-distance migrants, and (e,f) long-distance migrants. Results are from the breeding (a, c, e) and wintering (b, d, f) seasons. We used the best spatial models to derive these results. We excluded regions where the values of explanatory variables from the models were not within the analysis range (i.e., annual mean temperature < 2°C, elevation > 1600 m, snow depth > 2.4 m). Coordinates: 30°59′–45°31′N; 129°33′–145°49′E.


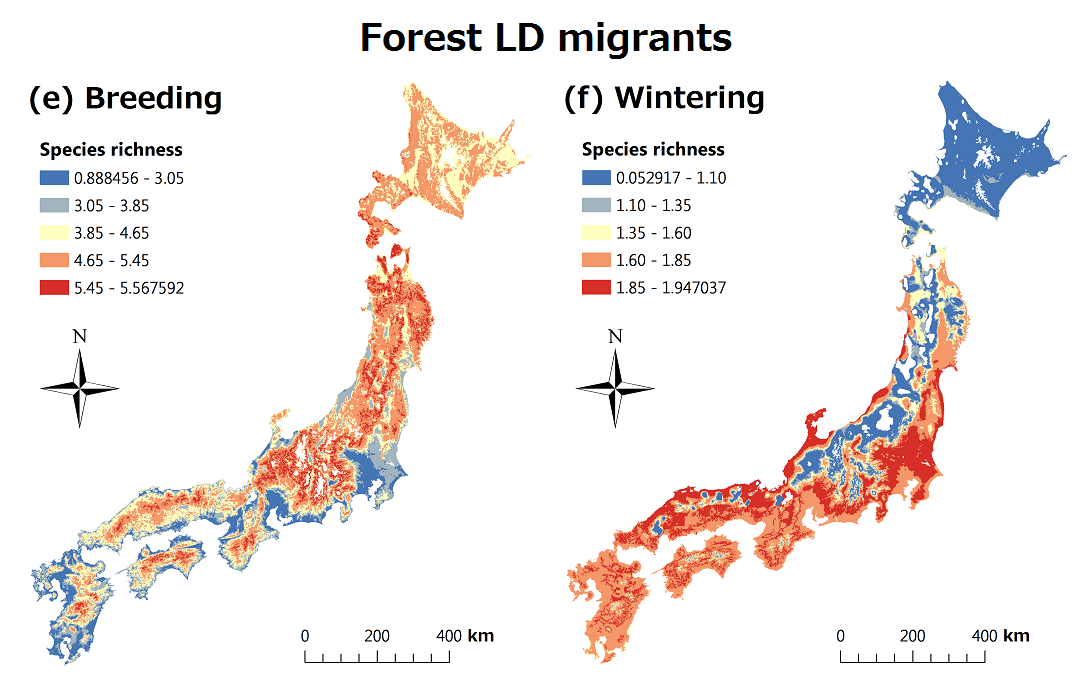


Appendix S13, continued.


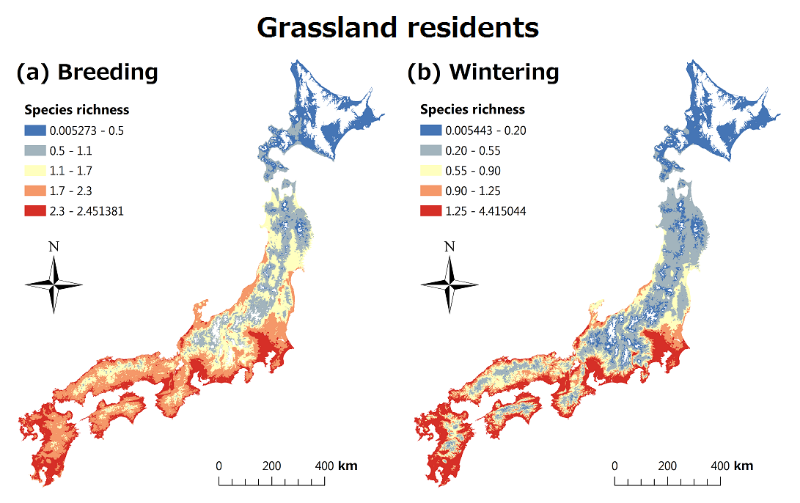


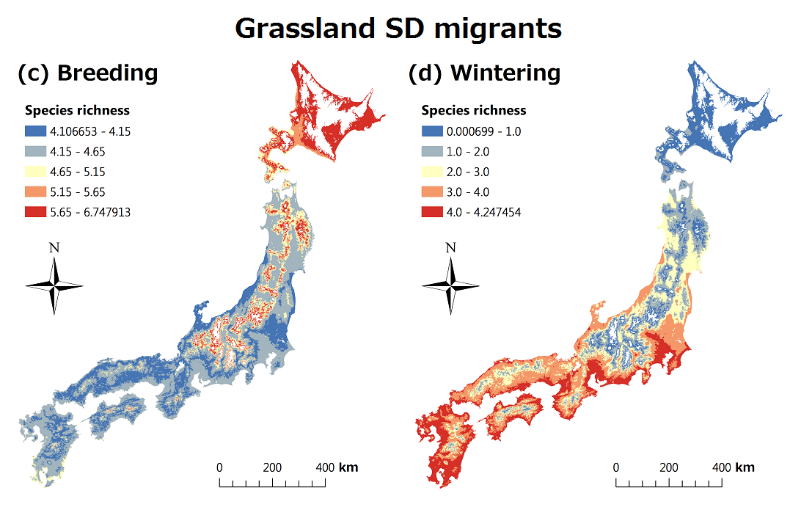


Appendix S14. Expected species richness of grassland (a–b) residents, (c–d) short-distance migrants, and (e–f) long-distance migrants. Results are from the breeding (a, c, e) and wintering (b, d, f) seasons. We used the best spatial models to derive these results. We excluded regions where the values of explanatory variables from the models were not within the analysis range (i.e., annual mean temperature <5 º C, elevation > 1600 m, snow depth > 2.4 m). Coordinates: 30°59′–45°31′N; 129°33′–145°49′E.


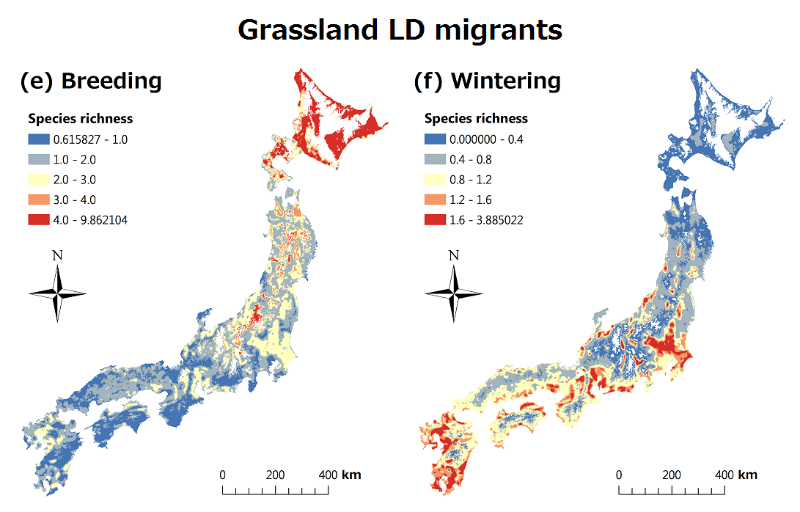


Appendix S14, continued.


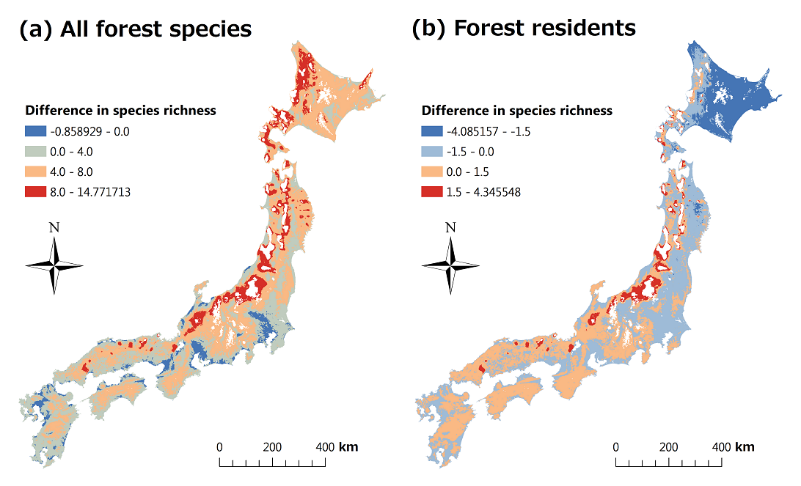


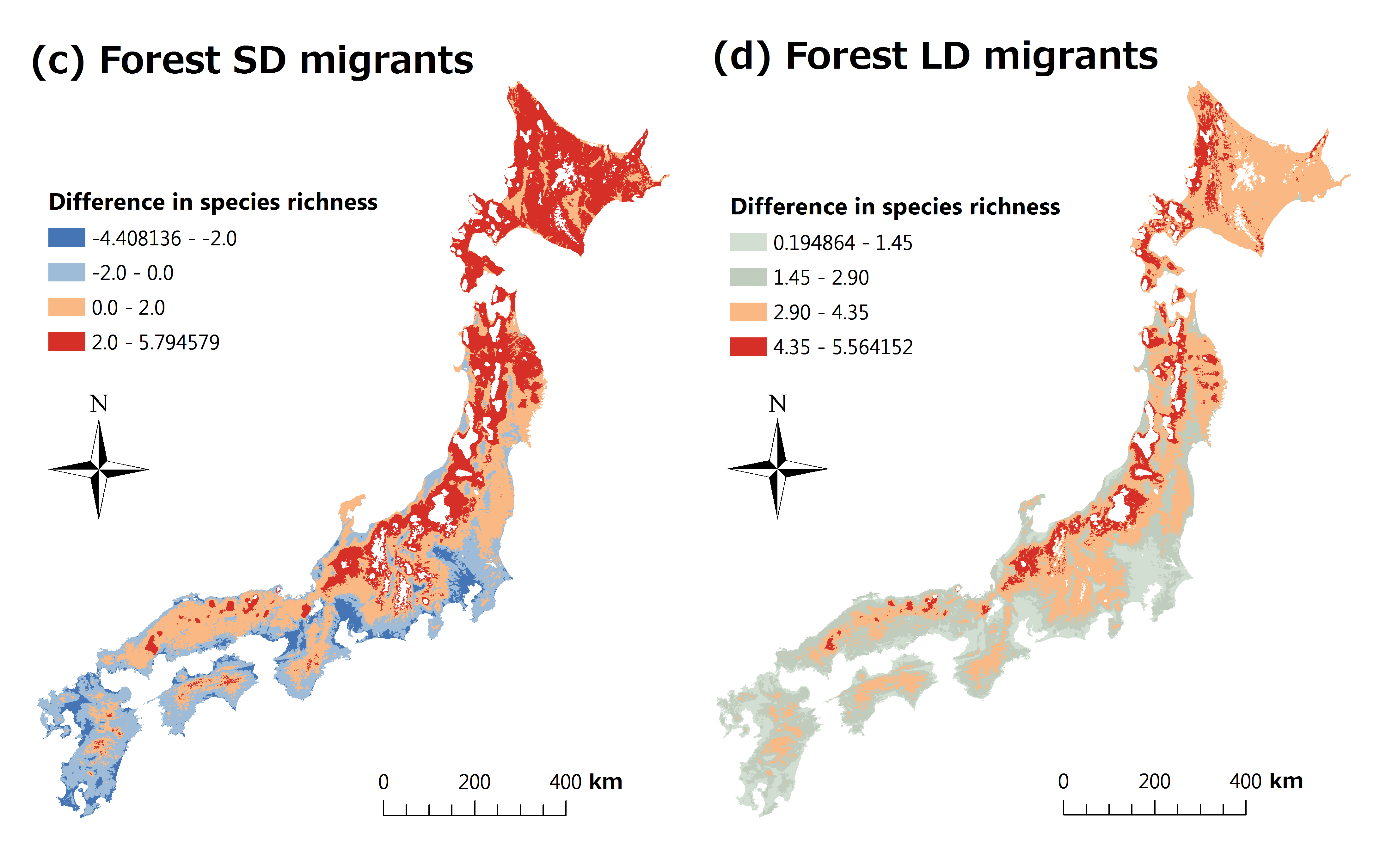


Appendix S15. Differences in expected species richness of forest birds between the breeding and wintering seasons [(a) all species, (b) residents, (c) short-distance migrants, and (d) long-distance migrants]. A positive value indicates that species richness was greater in the breeding than in the wintering season. We used the best spatial models to derive these results. We excluded regions where the values of explanatory variables from the models were not within the analysis range (i.e., annual mean temperature < 2°C, elevation > 1600 m, snow depth > 2.4 m). Coordinates: 30°59′–45°31′N; 129°33′–145°49′E.


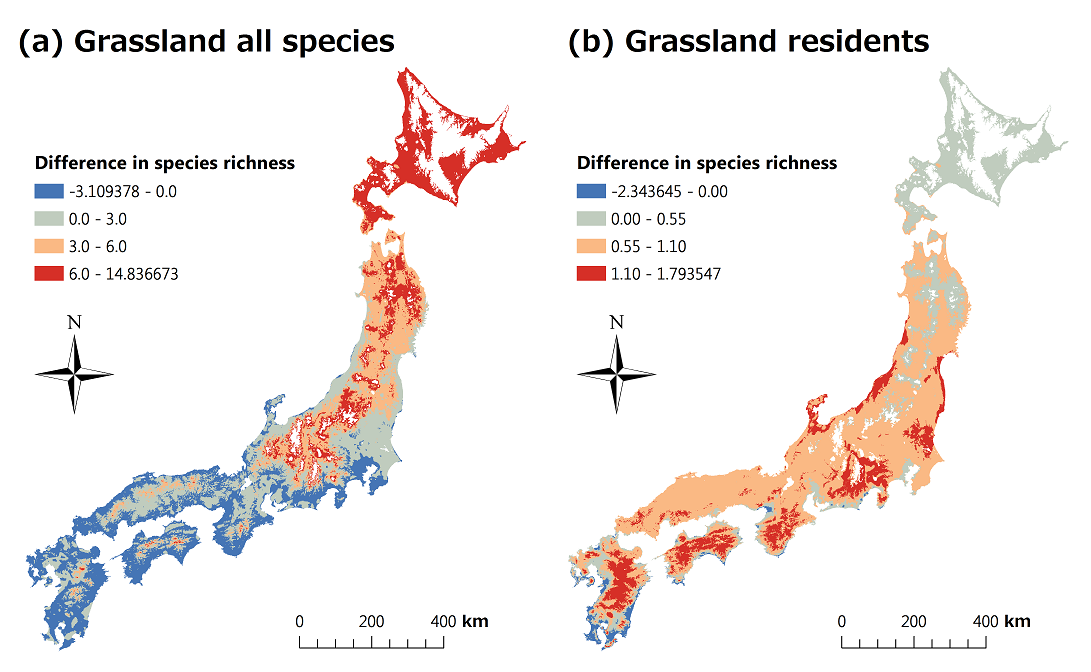


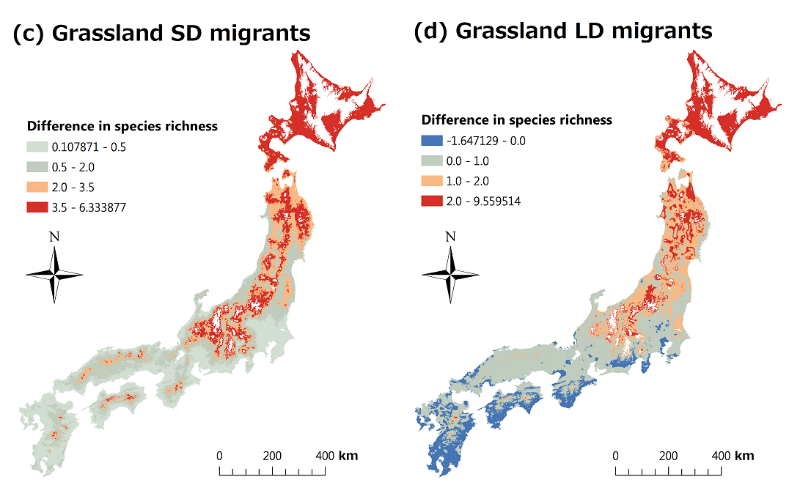


Appendix S16. Difference in expected species richness of grassland birds between the breeding and wintering seasons [(a) all species, (b) residents, (c) short-distance migrants, and (d) long-distance migrants]. A positive value indicates that species richness was greater in the breeding than in the wintering season. We used the best spatial models to derive these results. We excluded regions where the values of explanatory variables from the models were not within the analysis range (i.e., annual mean temperature < 5°C, elevation > 1600 m, snow depth > 2.4 m). Coordinates: 30°59′–45°31′N; 129°33′–145°49′E.
